# Light-Control over Casein Kinase 1δ Activity with Photopharmacology: A Clear Case for Arylazopyrazole-Based Inhibitors

**DOI:** 10.3390/ijms23105326

**Published:** 2022-05-10

**Authors:** Albert M. Schulte, Dušan Kolarski, Vidya Sundaram, Ashutosh Srivastava, Florence Tama, Ben L. Feringa, Wiktor Szymanski

**Affiliations:** 1 Stratingh Institute for Chemistry, University of Groningen, 9747 AG Groningen, The Netherlands; a.m.schulte@rug.nl (A.M.S.); kolarski@dwi.rwth-aachen.de (D.K.); 2 Discipline of Biological Engineering, Indian Institute of Technology Gandhinagar, Gandhinagar 382355, India; vidya.s@iitgn.ac.in (V.S.); ashutosh.s@iitgn.ac.in (A.S.); 3 Institute of Transformative BioMolecules (WPI-ITbM), Nagoya University, Chikusa, Nagoya 464-8601, Japan; florence.tama@nagoya-u.jp; 4 Department of Physics, Graduate School of Science, Nagoya University, Chikusa, Nagoya 464-8602, Japan; 5 Computational Structural Biology Unit, RIKEN-Center for Computational Science, Chuo, Kobe 650-0047, Japan; 6 Department of Radiology, Medical Imaging Center, University Medical Center Groningen, University of Groningen, 9713 GZ Groningen, The Netherlands

**Keywords:** photopharmacology, kinases, molecular photoswitches, arylazopyrazole photoswitches

## Abstract

Protein kinases are responsible for healthy cellular processes and signalling pathways, and their dysfunction is the basis of many pathologies. There are numerous small molecule inhibitors of protein kinases that systemically regulate dysfunctional signalling processes. However, attaining selectivity in kinase inhibition within the complex human kinome is still a challenge that inspires unconventional approaches. One of those approaches is photopharmacology, which uses light-controlled bioactive molecules to selectively activate drugs only at the intended space and time, thereby avoiding side effects outside of the irradiated area. Still, in the context of kinase inhibition, photopharmacology has thus far been rather unsuccessful in providing light-controlled drugs. Here, we present the discovery and optimisation of a photoswitchable inhibitor of casein kinase 1δ (CK1δ), important for the control of cell differentiation, circadian rhythm, DNA repair, apoptosis, and numerous other signalling processes. Varying the position at which the light-responsive azobenzene moiety has been introduced into a known CK1δ inhibitor, LH846, revealed the preferred regioisomer for efficient photo-modulation of inhibitory activity, but the photoswitchable inhibitor suffered from sub-optimal (photo)chemical properties. Replacement of the bis-phenyl azobenzene group with the arylazopyrazole moiety yielded a superior photoswitch with very high photostationary state distributions, increased solubility and a 10-fold difference in activity between irradiated and thermally adapted samples. The reasons behind those findings are explored with molecular docking and molecular dynamics simulations. Results described here show how the evaluation of privileged molecular architecture, followed by the optimisation of the photoswitchable unit, is a valuable strategy for the challenging design of the photoswitchable kinase inhibitors.

## 1. Introduction

Casein kinase 1 (CK1) protein kinases are members of the serine/threonine-specific protein kinase superfamily acting as monomeric enzymes and exclusively employing ATP as an energy-rich phosphate donor [1]. In eukaryotes, from yeast to humans, kinase domains of the CK1 isoforms have been evolutionary highly conserved [2]. The CK1 family regulates numerous signal transduction pathways, and in nearly all mammalian cell types it plays an essential regulatory role in controlling cell differentiation, proliferation, chromosome segregation, DNA repair, apoptosis, Wnt signalling, and circadian rhythms [2,3,4]. So far, at least seven CK1 isoforms (α, β, γ1, γ2, γ3, δ and ε) and various post-translationally modified splice variants have been discovered and characterised in humans [1,3]. Their abundance and critical role in numerous signalling processes explain why deregulation or dysfunction of CK1 contributes to pathological conditions such as tumorigenesis (overexpression of CK1α/δ/ε) [3], Alzheimer’s disease (upregulation of CK1δ) [2], familial advanced sleep phase syndrome (mutation of CK1δ/ε) [5], neurodegenerative diseases (CK1δ) [6], and inflammation (overexpression of CK1α/δ/ε) [7,8]. For that reason, there is a need to discover new and selective small molecules that modulate CK1 activity with a high level of specificity.

This work focuses on developing a photo-responsive modulator of CK1δ activity, as this isoform is linked to various pathologies [7,8]. The discoveries of the ATP-competitive inhibitors of CK1δ/ε were so far focused on obtaining a target selectivity towards these isoforms [9]. However, once the target-selectivity has been achieved, the quest to obtain locally activated inhibition remains. Local activation of the drug would suppress the potential side effects due to systemic CK1δ inhibition throughout the organism, and in addition, it would allow for more precise dissection of signalling pathways during fundamental studies concerning the site-specific role of kinases. Photopharmacology, an emerging research field that lies at the crossroad of medicinal chemistry, photochemistry, and chemical biology, enables the control of the activity of small molecules with light and high spatial and temporal precision [10,11,12,13]. For this purpose, photopharmacology relies on the modification of the known bioactive compounds with a broad palette of molecular photoswitches [14], such as diarylethenes [15,16], stilbenes [17], diazocines [18,19], fulgimides [20], and finally, azobenzenes, which represent the most studied and applied class of tools for photopharmacology [21,22]. Strategies to render small molecule photo-responsive with azobenzenes are converting a part of the parent molecule into an azobenzene (azologisation) or extending the structure with an arylazo moiety (azo-extension) [21,23,24].

**Figure 1:**
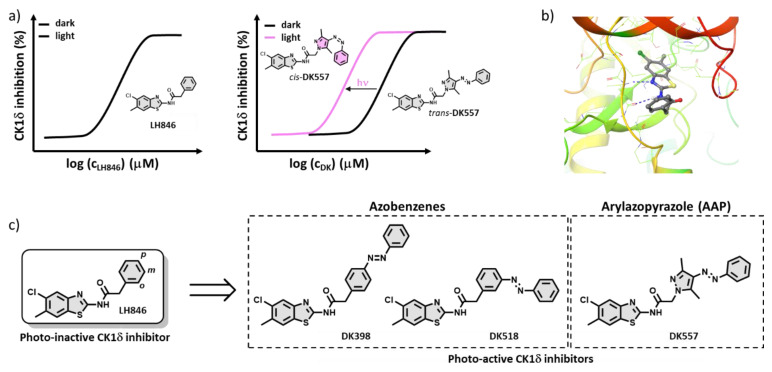
Design of the photoswitchable CK1δ inhibitor. (**a**) The concept of light-activated CK1δ inhibition; (**b**) molecular docking of **LH846** with CK1δ isoform; (**c**) the original CK1δ-selective inhibitor **LH846** was rendered photo-active by azo-extension in *para* (*p*, **DK398**) and *meta* (*m*, **DK518**) positions and by substituting the azobenzene moiety with the AAP photoswitch (**DK557**).

Despite the fact that the first photopharmacological small molecule kinase inhibitors were reported already in 2015 [25], a reversible control of kinase activity has been an ongoing effort rewarded with limited success [16,19,26,27,28,29,30,31,32,33,34]. The difficulty in obtaining a functional photoswitchable kinase inhibitor, especially by introducing photoswitches from the azobenzene class, comes from the large number of (photo)chemical parameters that need to be optimised to achieve a high level of photocontrol with retention of the potency, solubility, and stability of the parent bioactive compound. The parameters that are essential to achieve a significant difference in activity between the *trans*-(stable isomer) and *cis*-form (metastable isomer) include large geometrical changes upon the light-exposure, long half-life of the *cis*-form and photostationary state distributions (PSD) around 80–90% [31]. Moreover, chemical stability and solubility have emerged as persistent challenges throughout the development of new photoswitchable kinase inhibitors [19,32,33,35]. Some of the azo-modified kinase inhibitors suffered from the chemical instability of the azo bond due to nitrogen-rich and electron-poor structures of the parent kinase inhibitors that mimic the purine moiety of ATP [36]. As a result, the azo-group was converted to the corresponding hydrazine by the reducing agents present in the kinase assay buffers (e.g., dithiothreitol—DTT) or in cells (e.g., glutathione—GSH) [32,33]. The reduction of the azo-group can be circumvented by avoiding a direct conjugation of the azobenzene with the nitrogen-rich heteroaromatic moiety upon azologisation and finding a suitable aromatic position for the azo-extension [32−34]. Furthermore, azo-extension relies on the introduction of an additional arylazo moiety that can negatively impact solubility compared to the parent molecule due to its nonpolar and flat nature [19,33]. Furthermore, most of the existing azobenzene-based kinase inhibitors show higher potency towards the target in their thermally stable *trans*-form, while upon irradiation, the *cis*-enriched PSD has a lower potency (undesirably switching the activity OFF with light) [25,29,30,31]. The last challenge becomes essential during longer and spatially precise in cellulo, ex vivo and in vivo experiments. If the *trans*-isomer is biologically more active, the inhibitory effect is often not entirely abolished upon light irradiation due to incomplete *trans*-to-*cis* photoisomerisation. Furthermore, the thermal *cis*-to-*trans* isomerisation decreases the spatial and temporal resolution caused by reactivation. While it is possible to resolve the last challenge by designing a thermally stable photoswitch with nearly quantitative PSD [31], ideally, the *cis*-isomer should be designed to exhibit a higher potency as presented in this work and several previous cases (vide infra, Figure 1a) [12].

These challenges explain the limited success in developing photoswitchable CK1 (α, δ, ε) kinase inhibitors [32,33], with only one example reversibly modulating the CK1 activity in vitro, in cells, and ex vivo [31]. In this work, we present the design, preparation and in vitro analysis of a photoswitchable CK1δ inhibitor with quantitative photoisomerisation, high (photo)chemical stability, optimised solubility and a 10-fold difference in potency between the dark (all-*trans*) and irradiated (*cis*-enriched) sample, the attributes of which were rationalised using computational studies. The successful design of this photopharmacological drug was enabled by the use of a special subtype of the heteroaromatic azobenzenes [37], an arylazopyrazole (APP) photoswitch, introduced by the Fuchter group in 2014 [22], and studied in detail in the following years [38,39,40,41].

## 2. Results

### 2.1. Design of the Photoswitchable Inhibitors

The starting point in developing a photoswitchable CK1δ inhibitor was the work of Lee et al. [42], where a high-throughput screening campaign yielded compound **LH846** as a potent and selective inhibitor of CK1δ (Figure 1). The core structure of the **LH846** inhibitor is based on the 2-amido-benzothiazole scaffold, similar to recently published selective ATP-competitive CK1δ/ε inhibitors [9]. The structure–activity relationship (SAR) study identified that the benzyl amide’s para-position (*p*, Figure 1c) could be derivatised with a long and linear PEG-chain of the affinity probe without a significant loss in activity [42]. In the absence of the CK1δ-**LH846** crystal structure, we performed molecular docking (Figure 1b) and supported our results with the crystal structure of similar 2-amido-benzothiazole inhibitors bound to CK1δ [9]. The benzothiazole moiety is located in the hinge region of the ATP-binding site, and the recognition involves a hydrogen bond between the amide group, the benzothiazole’s nitrogen and Leu85, as well as van der Waals interactions between benzothiazole and Ile23 and Leu85 (Figure 1b) [40]. For that reason, we have decided to retain the 2-amido-benzothiazole core in the design of the photoswitchable variant, due to its importance for binding with the hinge region. This prompted us to avoid an intuitive benzyl amide azologisation that, as previously discussed, has resulted in susceptibility towards reduction in some of the earlier photoswitchable CK1 inhibitors (vide ante) [32,33]. On the other hand, the benzyl group is solvent-exposed, and fine-tuning of CK1δ/ε inhibition has been previously achieved by introducing subtle changes at *o-*, *m-*, and *p*-positions [9,42]. Besides the aforementioned introduction of a PEG-chain in the *p*-position by Lee et al. [42], García-Reyes et al. tuned the inhibitory activity with different substituents on the benzyl moiety [9], that led us to the conclusion that azo-extension at the benzyl group would be optimal. We envisioned that significant geometrical changes due to *trans*-to-*cis* photoisomerisation would reflect on the inhibition of the designed photoswitchable CK1δ inhibitors. Accordingly, the benzyl group was extended with the phenylazo-moiety in *p*- and *m*-positions (**DK398** and **DK518**, respectively, Figure 1c). The decision to incorporate the AAP scaffold (**DK557**, Figure 1c) was driven by the need to enhance solubility and photochemical properties of the photo-responsive inhibitor, as discussed after. AAP photoswitches are known to exhibit almost quantitative PSDs, better solubility, long half-lives. and higher photoisomerisation quantum yields than the diphenyl-azobenzenes [22].

### 2.2. Synthesis

**Scheme 1:**
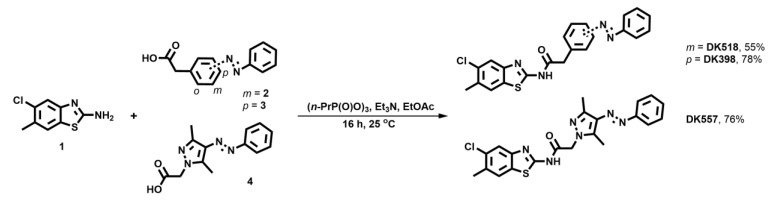
Synthesis of photoswitchable CKIδ inhibitors **DK398**, **DK518**, and **DK557**.

All three photoswitchable inhibitors were prepared in the coupling reaction between a commercially available 5-chloro-6-methylbenzo[*d*]thiazol-2-amine (**1**) and the corresponding azo-acids (**2–4**) using propanephosphoric acid cyclic anhydride (*n*-PrP(O)O)_3_ as an acid-activating reagent (Scheme 1) [42]. The azo-acids **2** and **3** were obtained by following published procedures [43,44].

### 2.3. Photochemical Evaluation

**Figure 2:**
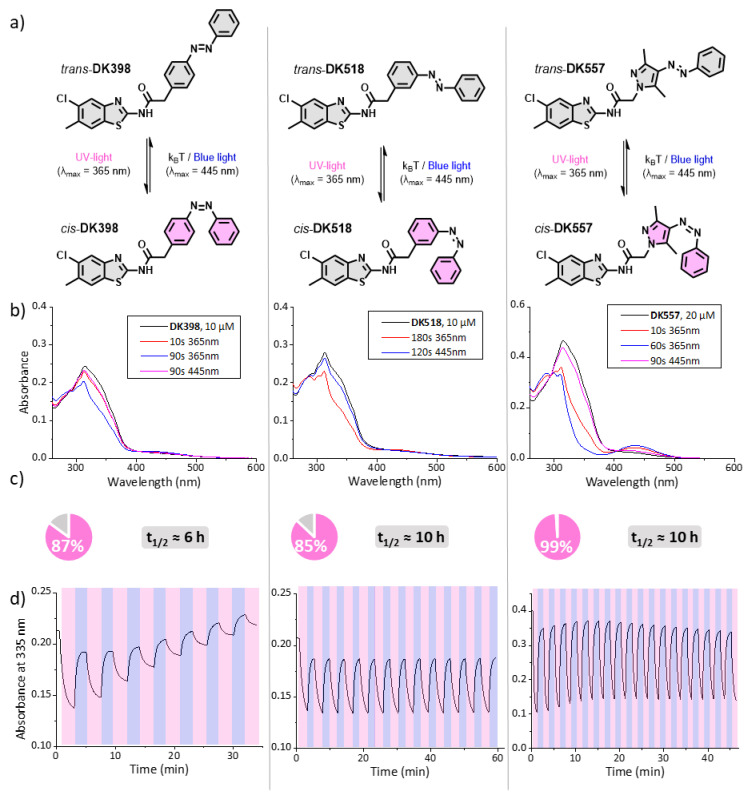
(**a**) Structures and irradiation-dependent interconversion of the *trans* and *cis* isomers of **DK398**, **DK518**, and **DK557**; (**b**) UV-Vis absorption spectra of **DK398**, **DK518**, and **DK557** in kinase assay buffer (10–20 µM, 4.8% DMSO) in the thermally adapted (all *trans*) and irradiated forms; (**c**) isomer distribution in the photostationary state (pie charts). The pink region represents the percentage of *cis*-isomer in the PSD as determined by ^1^H-NMR (DMSO-*d_6_*). The half-life for the thermal *cis*-to-*trans* isomerisation reaction (20 µM, kinase assay buffer, 1% DMSO, 35 °C) (Figure S4); (**d**) reversible switching cycles in kinase assay buffer containing DTT (0.5 mM) using UV light (λ_max_ = 365 nm) and blue light (λ_max_ = 445 nm).

With the synthesised azo-**LH846** analogues **DK398**, **DK518**, and **DK557** in hand, we set out to study their photochemical properties. UV-Vis spectrophotometry revealed that all three compounds displayed reversible photochromism in the buffer used in the kinase activity assay (Figure 2a,b) and in DMSO (Figures S1–S3). Samples were thermally adapted before the measurement, to yield the pure *trans* isomer as the starting point (Figure 2b, black lines). All photoswitches showed the expected π-π* transition band around 300–370 nm. Irradiation of this band with UV-light (λ_max_ = 365 nm) led to the formation of a *cis*-enriched photostationary state (PSS) and emergence of a new n-π* band around 400–500 nm. Irradiation of this band with blue light (λ_max_ = 445 nm) resulted in back-isomerisation to a *trans*-enriched state.

^1^H-NMR analysis of the samples under irradiation confirmed the reversible formation of the *cis-*isomer (Figures S5–S7). After 2 h of irradiation, the isomer distribution in the PSS was determined. All compounds showed a considerable fraction of the *cis* isomer in the PSD (Figure 2c), with **DK557** showing near quantitative (>99%) conversion under irradiation. Furthermore, all compounds displayed a low rate for the thermal *cis-*to-*trans* isomerisation, featuring half-lives ranging from 5 to 10 h in the kinase assay buffer (Figure 2c and Figure S4).

Lastly, the chemical stability of the photoswitches was evaluated in the kinase assay buffer. To avoid the oxidation of the kinase substrate peptide, the buffer contains dithiothreitol (DTT) as a reducing agent. Therefore, we presumed that performing multiple switching cycles in this buffer would allow us to gain insight into both the photochemical- and chemical stability of the three photoswitches. While **DK518** and **DK557** showed excellent stability over multiple switching cycles, **DK398** displayed poor stability (Figure 2d). LCMS analysis revealed that all compounds were stable against reduction by DTT (Figure S8); therefore, the observed instability of **DK398** must have other causes. We hypothesise that the instability of **DK398** in the assay buffer was caused due to either poor solubility or interactions with buffer components, such as the BSA protein.

### 2.4. Light Modulation of CK1δ Activity

After evaluating the photochemical properties of the three photoswitches **DK398**, **DK518**, and **DK557** in detail, an in vitro kinase activity assay was performed [42]. In this assay, the inhibitory activity towards CK1δ of both dark (all-*trans*) and irradiated (*cis*-enriched) samples was evaluated to construct dose–response curves for both states. During the enzymatic reaction (2 h), samples were either kept in the dark or irradiated with UV light (λ_max_ = 365 nm). UV light irradiation itself was confirmed to have no effect on CK1δ activity (Figure S9). After the enzymatic reaction, ATP consumption was measured to determine enzyme activity. The ATP consumption of the samples containing the photoswitchable inhibitors was compared to the ATP consumption of a DMSO control to assess the degree of enzyme inhibition.

Initially, we set out to study the CK1δ inhibition of the different geometries of *para-* and *meta-*substituted azobenzenes **DK398** and **DK518**. Constitutional isomers of azobenzene-based photoswitchable inhibitors can give rise to remarkably different irradiation-dependent inhibitory activities of their target enzymes [31]. By comparing the two designs, we aimed to find a photoswitch architecture that showed irradiation-dependent enhancement of its inhibitory activity.

**Figure 3:**
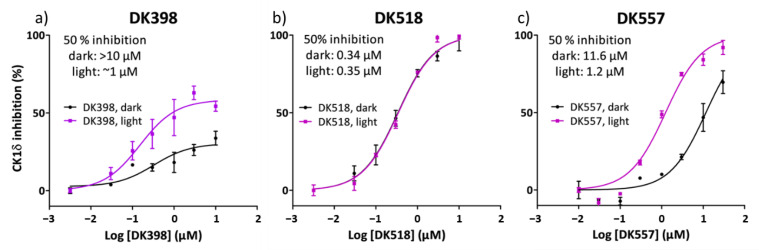
Dose–response curves displaying a degree of CK1δ inhibition for ‘dark’ (black) and irradiated samples (purple, ‘light’) of the photoswitchable inhibitors (**a**) **DK398**, (**b**) **DK518**, and (**c**) **DK557**. ‘Light’ samples were irradiated with UV light (λ_max_ = 365 nm) for the full duration of the assay (2 h). Shown are averages and standard deviations of triplicate samples.

To our satisfaction, upon irradiation, the *para*-substituted azobenzene **DK398** displayed a notable difference in inhibitory activity (Figure 3a). Whereas, in the dark, a concentration of >10 µM was required for 50% inhibition of CK1δ; upon irradiation, the required concentration decreased to ~1 µM, constituting approximately a 10-fold difference in activity. However, for **DK398** neither the ‘light’ nor ‘dark’ state accomplished full inhibition of CK1δ activity. To still make a fair comparison between inhibitory activities of the three photoswitchable **LH846** analogues, we decided to compare their absolute rather than relative IC_50_ values.

We hypothesised that the incomplete inhibition of CK1δ by **DK398** in the kinase assay buffer could at least be partially explained by the observed poor stability of **DK398** (Figure 2d), rather than by the actual poor inhibitory activity of the stereoisomers. Similar behaviour has already been linked to the poor solubility of photo-responsive kinase inhibitors [19,33]. Given the higher stability of *meta*-substituted azobenzene **DK518** (Figure 2d), we were interested to learn if this property would be reflected in its dose–response curve. Indeed, in contrast to **DK398**, both ‘light’ and ‘dark’ samples of **DK518** achieved complete enzyme inhibition (Figure 3b). Furthermore, **DK518** displayed high potency towards CK1δ, showing IC_50_ values of ~0.35 µM, comparable to the IC_50_ of **LH846** (0.29 µM) [42]. As hypothesised, this illustrates that azo-extension at the phenyl moiety of **LH846** is well tolerated and results in compounds that still display high inhibitory activities towards CK1δ. Unfortunately, despite the increased degree of CK1δ inhibition, *meta-*substitution in **DK518** completely abolished the difference in activity between the ‘light’ and ‘dark’ states observed for **DK398**. These results illustrate the tight connection between the position of azo-extension and the inhibitory activity of the *cis* and *trans* stereoisomers, with the two constitutional isomers **DK398** and **DK518** showing such distinct irradiation-dependent inhibitory activities.

From the comparison between the bioactivity data of **DK398** and **DK518**, we learned that *para*-substitution was required to achieve the desired difference in inhibitory activity. However, the instability of **DK398** in the assay buffer led to its poor overall inhibitory activity. In search of a more stable photoswitch, we arrived at **DK557**, an aryl azopyrazole known to have superior solubility and improved photochemical properties. Furthermore, for **DK557**, the computational analysis revealed that the geometry of **DK557** showed a closer resemblance to *para*-substituted **DK398** than to *meta*-substituted **DK518** (Supporting Figure S10). Therefore, we hypothesised that this geometrical similarity would also be translated into an irradiation-dependent increase in inhibitory activity for **DK557**, as was observed for **DK398**. To our delight, irradiation resulted in a significant increase in the inhibitory activity of **DK557** (Figure 3c). Similar to **DK398**, the ‘light’ state showed approximately a 10-fold higher potency than the ‘dark’ state. Furthermore, in contrast to **DK398**, **DK557** did achieve complete enzyme inhibition upon irradiation. This effect could be explained by the higher solubility of AAP photoswitches as compared to azobenzenes, corresponding with the stability of AAP **DK557** in the assay buffer. In our case, the AAP photoswitch **DK557** combines the best of both worlds: high stability in the assay buffer resulting in a high degree of enzyme inhibition, and a significant difference between the ‘light‘ and ‘dark’ states.

### 2.5. Molecular Docking and Dynamic Simulations

**Figure 4:**
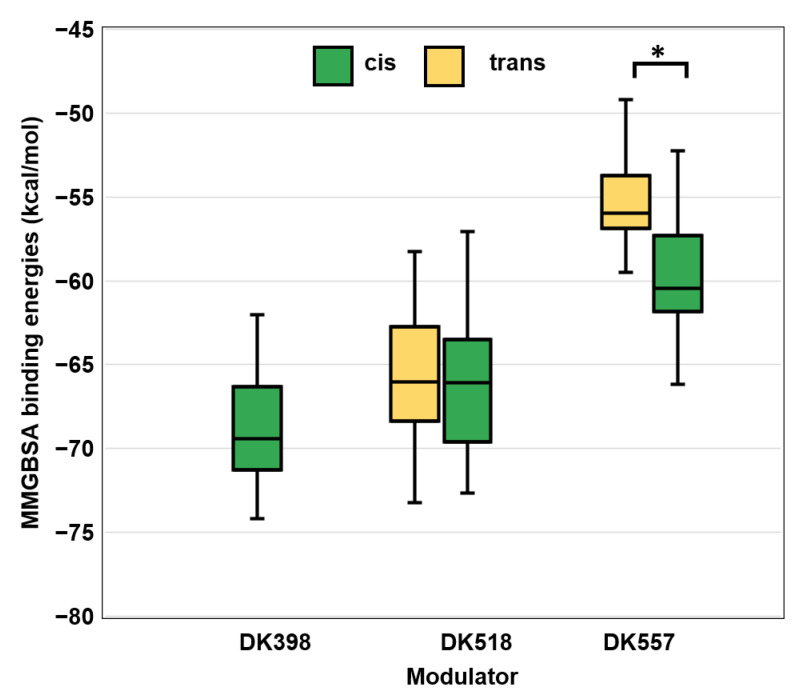
Box-and-whisker plot of the MM-GBSA binding energies of the photoswitchable molecules **DK398, DK518**, and **DK557** with CK1δ. The *trans* form of **DK398** was not considered for MM-GBSA analysis due to its poor docking score. The interquartile range of binding free energies is shown in the bounded box with the horizontal line within the box denoting the median. The whiskers show the entire range of the binding free energies with the horizontal lines at the top and bottom representing the highest and lowest binding free energies, respectively. No significant difference in mean values between **DK518** *cis* and *trans* forms was found (Student’s *t*-test, *p* > 0.05), whereas, for **DK557** a significant difference was found (Student’s *t*-test, *p* < 0.05 or *) (for further statistical data, see SI Figure S14).

Molecular docking predicted that **DK398**, **DK518**, and **DK557** were all bound to the ATP-binding site and established the canonical hydrogen bond with Leu85 of CK1δ. However, the *trans* form of **DK398** established only a single hydrogen bond with Leu85 (Supporting Figure S11a) and displayed the weakest interaction with the lowest Glide score (G-score) of −1.74. Expectedly, molecular dynamics (MD) simulations revealed that the single hydrogen bond interaction between the *trans* form of **DK398** and Leu85 was lost even during the initial stages of the simulation, denoting a less stable complex of CK1δ and the *trans* form of **DK398**. In comparison, the *cis* form showed tighter binding with a more favourable G-score of −9.03; it formed two hydrogen bond interactions with Leu85 that remained stable throughout the course of the MD simulations (Supporting Figure S11b). These computations correspond well to the kinase activity assay results, which showed a higher potency of the ’light’ state of **DK398** compared to its ‘dark’ state (Figure 3a). In the case of **DK518** and **DK557**, both *trans* and *cis* forms showed the binding of two canonical hydrogen bonds with Leu85 (Figures S12 and S13). The molecular mechanics/Generalized Born surface area (MM-GBSA) binding energies of the *trans* and *cis* forms of **DK518** in complex with CK1δ were found to be similar (Figure 4), providing a computational explanation for the equal potencies of the ‘light’ and ‘dark’ states of **DK518** that were observed in the kinase assay (Figure 3b). In contrast, for AAP-functionalised inhibitor **DK557**, there was a considerable difference between the calculated binding energies of the *trans* and *cis* forms. The *cis* form of **DK557** showed lower binding energy as compared to the *trans* form (Figure 4), which is in agreement with the observed higher potency of the ‘light’ samples in the kinase assay (Figure 3c). In summary, these molecular docking and simulations provide a computational explanation for the observed irradiation-dependent effects in inhibitory activity for all three photoswitchable CK1δ inhibitors.

## 3. Material and Methods

**Organic synthesis**. All chemicals were purchased from Sigma–Aldrich (St. Louis, MO, USA), Acros (Thermo Fisher Scientific, Fisher Scientific The Hague IV B.V.), Fluka (Buchs, Switzerland), and TCI (Tokyo, Japan) and were used as received. An MBraun SPS-800 solvent purification system was used to obtain dry DCM, and aqueous solutions were prepared with deionised water. TLC analysis was performed on F254 silica gel plates with fluorescence-indicator UV254 (Merck, Darmstadt, Germany, Kieselgel 60) and the UV-active compounds were detected using UV light (254 nm or 365 nm). For the additional visualisation, oxidative staining of TLC plates with aqueous basic potassium permanganate solution (KMnO_4_) or aqueous acidic cerium phosphomolybdic acid solution (Seebach’s stain) was used. Organic solutions were dried over MgSO_4_ and solvents were removed in vacuo (Büchi, R-300). Detailed synthetic procedures are given in the Supporting Information (SI) File.

**Analytical procedures**. ^1^H NMR, ^13^C NMR, and ^19^F NMR spectra were recorded at room temperature (22–24 °C) on a 400 MHz Agilent Technologies 400-MR (400/54 Premium Shielded) spectrometer. As an internal reference, residual solvent peaks were used [CDCl_3_: δ_H_ = 7.26 ppm; CDCl_3_: δ_C_ = 77.16 ppm; DMSO-*d_6_*: δ_H_ = 2.50 ppm; DMSO-*d_6_*: δ_C_ = 39.52 ppm]. High-resolution mass spectrometric measurements were performed using a Thermo scientific LTQ OrbitrapXL spectrometer with ESI ionization. Melting points were recorded using a Stuart analogue capillary melting point SMP11 apparatus.

**Photochemical evaluation.** UV-Vis absorption spectra were recorded on an Agilent 8453 UV-Visible spectrophotometer equipped with an irradiation set-up containing a 365 and 445 nm LED. Stock solutions of **DK398**, **DK518**, and **DK557** were prepared in DMSO (2.0 mM) and were diluted 100-fold in kinase assay buffer (40 mM Tris-HCl, 10 mM MgCl_2_, 0.5 mM DTT, 0.1 mg/mL BSA, pH 7.4, final 1% DMSO) or DMSO for spectroscopic characterisation. Reversible switching cycles were performed in a kinase assay buffer to evaluate the (photo)chemical stability. All three compounds were irradiated using UV light (λ_max_ = 365 nm) to perform *trans*-to-*cis* isomerisation and blue light (λ_max_ = 445 nm) for the back-reaction. The irradiation time was adjusted to the compound until the corresponding PSS was achieved.

**In vitro kinase assay**. Stock solutions (2.0 mM) of the photoswitchable **LH846** inhibitors were prepared in DMSO and thermally adapted by heating to c.a. 100 °C for 1 min. Dilutions were made in DMSO to reach 21-fold concentrated solutions of the desired concentrations to be tested in the assay. The assays were performed on white, solid-bottom 384-well plates and electronic, hand-held Gilson pipettes were used. The total volume for the reaction was 10.5 µL. All solutions were applied to the well plate in a dark room to prevent ambient-light-induced photo-isomerisation. Firstly, a solution of CK1δ and substrate peptide RKKKAEpSVASLTSQCSYSS (Anaspec Fermont, CA, USA, custom made) in a kinase assay buffer was aliquoted into the bottom of the wells (9 µL). Next, the 21-fold concentrated solutions of the compound (0.5 μL, final 4.8% DMSO) was pipetted into the bottom of the wells. Lastly, a solution of ATP in water (50 µM, 1 μL) was pipetted into the upper corners of each well (final concentration 4.8 µM). Final concentrations of CK1δ and the substrate peptide were 2 ng/µL and 50 µM, respectively. The enzymatic reaction was started by spinning down the plate (1800× *g*, 2 min). By employing this method, all reactions were started at the same time, minimising variance between different samples. During the assay, ‘light’ samples were irradiated with UV light (λ = 365 nm, UV lamp Spectroline, ENB-280/FE, 1 × 8 Watt) for the full duration of the assay (2 h). The wells of ‘dark’ samples were carefully covered with an aluminium sticker. After the incubation period (2 h at 30 °C), 10 μL Kinase Glo^®^ (Promega, Madison, WI, USA) was applied into the wells and the luminescent signal was recorded by a plate reader (BioTek Synergy H1). All measurements were carried out in triplicate. Enzyme activity was determined by comparing the ATP consumption of the samples containing the **LH846**-analogues to that of a DMSO control, set as 100% enzyme activity.

**Docking and Molecular Dynamics Simulations.***Cis* and *trans* forms of **DK398**, **DK518**, and **DK557** were docked to seven high resolution crystal structures of CK1δ using Glide module of Schrödinger (Schrödinger Release 2020-4: Glide, Schrödinger, LLC, New York, NY, USA, 2020) and high-scoring complexes were taken for further analysis (see Supplementary Information for further details). The stability of docked complexes was analysed using 20 ns molecular dynamics simulations in explicit TIP3P solvent with OPLS3e force field. The last 2.5 ns of the trajectory was used to extract 20 equally spaced frames and binding energy calculations were performed on these frames using MM-GBSA (Please see Supplementary Text for further details).

## 4. Conclusions

This work shows the successful design of a photoswitchable CK1δ kinase inhibitor, which resulted from a comparative analysis between the most commonly used azobenzenes and their heterocyclic analogue arylazopyrazole (AAP). In photopharmacology, the development of photoswitchable kinase inhibitors has proven challenging, and only a handful of successful examples have been published so far. Some of the problems that hamper the progress in this field are photo- and chemical-stability of a photoswitchable moiety, low aqueous solubility, non-quantitative photoisomerisation (low PSDs), insufficient thermal stability of the metastable isomer, and the requirement for the turn-ON type of photoswitchable kinase inhibitors. Some of these issues can prevent studying a light-induced effect on the kinase activity modulation entirely (fast reduction and thermal back-isomerisation, very low solubility), while the others influence the magnitude of the observed activity modulation (low photostationary state distribution of isomers, moderate thermal back-isomerisation, etc.).

The chosen strategy to build a photo-responsive kinase inhibitor was the azo-extension of a known CK1δ-selective inhibitor **LH846** (Figure 1a). Molecular docking, MD simulations, and crystal structure analysis of **LH846** and similar benzothiazoles with CK1δ revealed a solvent-exposed benzyl amide moiety as a potential place for azo-extension (Figure 1c). Incorporation of the phenylazo group yielded two regioisomers—*para* (**DK398**) and *meta* (**DK518**). Comparison between these two regioisomers showed that both compounds are stable under reductive conditions, but the *meta*-isomer has better solubility and photochemical properties in the kinase buffer (Figure 2). However, in the kinase assay, for **DK518**, light-induced modulation of inhibitory activity was not observed (Figure 3b). At the same time, **DK398**, despite incomplete inhibition, showed a significant difference in activity between the ‘light’ and ‘dark’ states (Figure 3a). Consequently, the azobenzene moiety has been replaced with the AAP photoswitch to improve solubility and photochemical properties. This heterocyclic photoswitch exhibits superior properties to azobenzenes, such as almost quantitative PSD and higher aqueous solubility, while being thermally and chemically stable. Photochemical investigation revealed that **DK557** has an exceptional PSD of 99%, which is very high when compared to **DK398**, which yielded 87% of the *cis*-isomer under irradiation (Figure 2c). Moreover, reversible photochromism in the kinase buffer containing DTT also confirmed high stability towards reduction (Figure 2d). The possibility of reaching much higher concentrations with **DK557**, which is more soluble in the in vitro assay than its classical azobenzene counterparts **DK398** and **DK518**, led us to conclude that AAP indeed has superior (photo)chemical properties to typically employed bis-phenyl azobenzenes. In vitro kinase assay revealed that **DK557** in both states (‘light’ and ‘dark’) reaches almost complete inhibition, and the observed difference between the irradiated sample (‘light’) and thermally adapted (‘dark’) was 10-fold, with the *cis*-enriched sample being more potent than the *trans* form (Figure 3c). This observation was rationalised by the molecular docking and simulations where the MM-GBSA binding energy for the *cis*-isomer was lower than for the corresponding *trans*-isomer (Figure 4).

In summary, in two successive steps—finding the right azobenzene architecture and optimising its (photo)chemical properties—we designed a well-soluble and (photo)chemically stable photoswitchable kinase inhibitor with an almost quantitative photostationary state distribution. Additionally, the development led to an inhibitor whose potency was enhanced ten times upon light exposure. This work contributes to the discovery of new photo-responsive kinase inhibitors and gives directions on how to stepwise obtain photoswitches with the desired photo- and chemical-properties.

## Data Availability

Not applicable.

## Supplementary Materials

The following supporting information can be downloaded at: https://www.mdpi.com/article/10.3390/ijms23105326/s1.
