# Supplementary material for: Light-Control over Casein Kinase 1δ Activity with Photopharmacology: A Clear Case for Arylazopyrazole-Based Inhibitors
Source: Int J Mol Sci. 2022 May 10;23(10):5326. doi: 10.3390/ijms23105326 (PMC9140716; doi:10.3390/ijms23105326)
Supplement: Supplementary file 1 [file ijms-23-05326-s001.zip › ijms-1702459-supplementary.pdf]

Light-control over Casein Kinase 1δ activity with photopharmacology: a clear case for arylazopyrazole-based inhibitors

Contents

Synthesis.. 2

Final compounds 2

Precursors... 4

NMR Spectra.. 7

UV-Vis absorption spectra... 11

Half-life measurements... 13

Photostationary state isomer distribution (PSD) 14

Reductive stability (DTT) 17

Irradiation effect on CK1δ activity.. 18

Molecular Docking.. 19

Synthesis

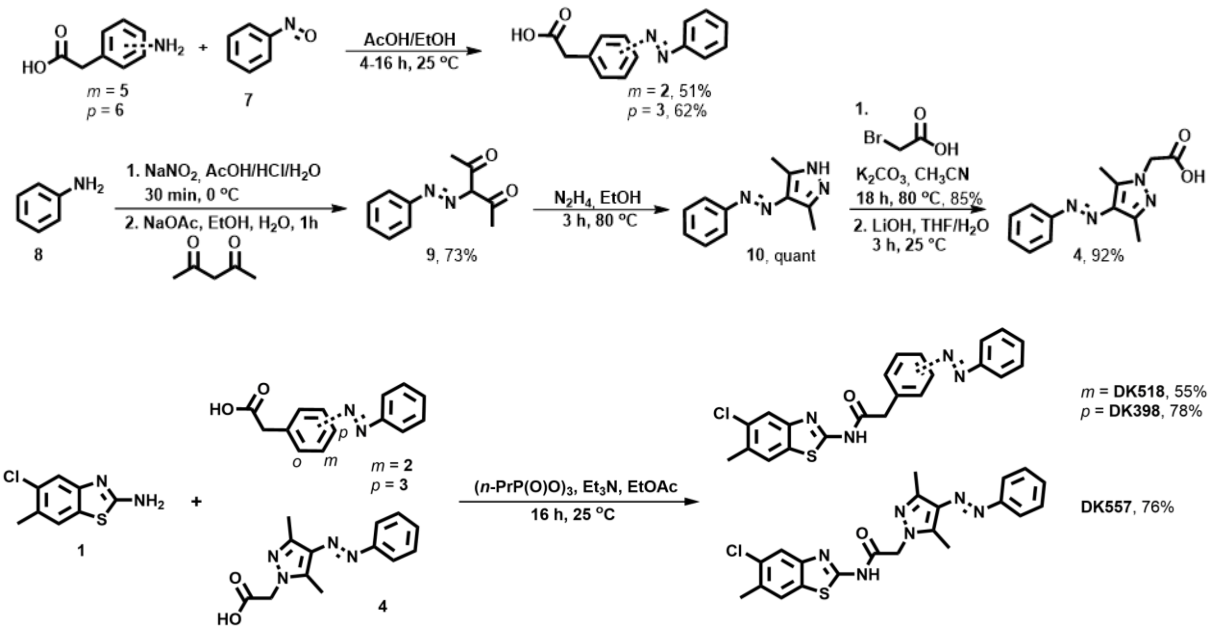

Final compounds

All final compounds were obtained in the coupling reaction between **1** and a corresponding azo-acid (**2**-**4**) following the literature procedure:[1]

A solution of 5-chloro-6-methylbenzo[d]thiazol-2-amine (**1**, 100 mg, 0.50 mmol, 1.0 eq.) and azo-acid (**2**-**4**, 0.65 mmol, 1.3 eq.) in EtOAc (5 mL) was treated with triethylamine (90 µL, 0.65 mmol, 1.3 eq.) and 50% (wt) propane phosphoric acid cyclic anhydride (*n*-PrP(O)O)$_3$ (386 µL, 0.65 mmol, 1.3 eq.) in EtOAc. The reaction mixture was allowed to stir overnight at room temperature and then diluted with CH_2Cl_2 and H_2O. The organic layer was extracted, dried over $MgSO_4$, filtered, and concentrated under reduced pressure. The product was purified by flash column chromatography (DCM/MeOH 98:2).

DK398

Beige solid. Yield 78%.

^{1}H NMR (400 MHz, DMSO-d_6) δ 7.93 (s, 1H), 7.87 (d, *J* = 8.3 Hz, 3H), 7.80 (s, 1H), 7.57 (t, *J* = 7.7 Hz, 6H), 3.95 (s, 2H), 2.39 (s, 3H) ppm;

^{13}C NMR (101 MHz, DMSO-d_6) δ 170.28, 159.24, 152.35, 151.35, 148.33, 138.71, 131.92, 131.90, 131.10, 130.96, 130.91, 129.87, 123.64, 123.08, 122.95, 120.62, 42.05, 20.22 ppm;

IR (ATR) $\tilde{\nu}$ 3123, 2951, 1694, 1530, 1447, 1279, 1117,1011, 807, 771, 721, 684 cm^{-1};

HRMS (ESI, $[M+H]^+$): calcd. for $C_{22}H_{18}ClNOS^+$: 421.0884 ; Found: 421.0886;

m.p. 244-246 °C.

DK518

Orange solid. Yield 55%.

^{1}H NMR (400 MHz, DMSO-d_6) δ 7.93 (s, 1H), 7.91 – 7.86 (m, 4H), 7.84 – 7.78 (m, 2H), 7.63 – 7.51 (m, 6H), 3.97 (s, 2H), 2.39 (s, 3H) ppm;

^{13}C NMR (101 MHz, DMSO-d_6) δ 170.51, 159.29, 152.40, 152.38, 148.35, 136.49, 133.01, 132.04, 131.92, 131.11, 130.92, 130.00, 129.94, 123.70, 123.45, 122.98, 122.28, 120.63, 42.00, 20.22 ppm;

IR (ATR) $\tilde{\nu}$ 3113, 3032, 2947, 1694, 1533, 1444, 1323, 1284, 1150, 1117, 1007, 889, 784, 687 cm^{-1};

HRMS (ESI, $[M+H]^+$): calcd. for $C_{22}H_{18}ClN_4OS^+$: 421.0884 ; Found: 421.0890;

m.p. 205-207 °C.

DK557

Yellow solid. Yield 76%.

^{1}H NMR (400 MHz, DMSO-d_6) δ 7.97 (s, 1H), 7.84 (s, 1H), 7.74 (d, *J* = 7.8 Hz, 2H), 7.52 (t, *J* = 7.6 Hz, 2H), 7.43 (t, *J* = 7.3 Hz, 1H), 5.20 (s, 2H), 2.57 (s, 3H), 2.39 (d, *J* = 8.4 Hz, 7H) ppm;

^{13}C NMR (101 MHz, $CDCl_3$) δ 165.15, 157.04, 153.26, 147.49, 145.34, 140.30, 135.62, 132.98, 132.47, 130.76, 129.99, 128.97, 122.45, 121.97, 121.29, 51.56, 20.43, 14.19, 9.89 ppm;

IR (ATR) $\tilde{\nu}$ 3180, 3004, 1702, 1605, 1571, 1506, 1449, 1423, 1376, 1280, 1180, 991, 865, 763, 7366, 682 cm^{-1};

HRMS (ESI, $[M+H]^+$): calcd. for $C_{21}H_{20}ClN_6OS^+$: 439.1102 ; Found: 439.1104;

m.p. 209-211 °C.

Precursors

DK398 precursors

To a solution of 2-(4-aminophenyl)acetic acid (**6**, 1.2 g, 7.9 mmol, 1.0 equiv) in acetic acid (8 mL) was added nitrosobenzene (**7**, 0.9 g, 8.4 mmol, 1.1 equiv). The mixture was stirred for 4 h at room temperature and filtered. The residue was washed with acetic acid (2x), water (1x) and dried under reduced pressure to yield **3** (1.18 g, 4.9 mmol, 62 %).

The spectra corresponds to the published data.[2]

^{1}H-NMR (400 MHz, DMSO-d_6) δ 7.85 (dd, J = 13.4, 7.9 Hz, 4H), 7.67 – 7.42 (m, 5H), 3.69 (s, 2H) ppm;

^{13}C-NMR (101 MHz, DMSO-d_6) δ 172.71, 152.37, 151.15, 139.31, 131.88, 130.98, 129.90, 122.94, 122.91, 40.87 ppm;

m.p. 183-184 °C.

DK518 precursors

2-(3-aminophenyl)acetic acid (**5**, 1.0 g, 6.6 mmol, 1 eq.) and nitrosobenzene (**7**, 921 mg, 8.6 mmol, 1.3 eq.) were dissolved in AcOH/EtOH (10:1, 60 mL) and stirred overnight at room temperature. Volatiles were removed and the product was purified by flash column chromatography (PhMe/EtOAc 1:1). The product was obtained as an orange solid (**2**, 0.81 g, 3.4 mmol, 51%).

^{1}H NMR (400 MHz, DMSO-d_6) δ 7.88 (d, *J* = 6.2 Hz, 2H), 7.80 – 7.76 (m, 2H), 7.64 – 7.50 (m, 4H), 7.45 (d, *J* = 7.5 Hz, 1H), 3.72 (s, 2H) ppm;

^{13}C NMR (101 MHz, DMSO-d_6) δ 172.90, 152.38, 152.32, 137.01, 133.08, 131.98, 129.93, 129.75, 123.53, 122.96, 121.85, 40.76.

IR (ATR) $\tilde{\nu}$ 3029, 2920, 2738, 1694, 1479, 1411, 1350, 1297, 1261, 1217, 1185, 905, 758, 688 cm^{-1};

HRMS (ESI, $[M+H]^+$): calcd. for $C_{14}H_{12}N_2O_2S^+$: 241.0972 ; Found: 241.0964;

m.p. 110-111 °C.

DK557 precursors

$NaNO_2$ (1.78 g, 25.8 mmol, 1.2 eq.) dissolved in a minimum amount of water was added dropwise to a solution of aniline (**8**, 2 g, 1.96 mL, 21.5 mmol, 1.0 eq.) in AcOH (30 mL) and HCl (12 M, 5 mL) at 0 °C. After stirring the reaction mixture for 45 minutes, the resulting diazonium salt was transferred to a suspension of pentane-2,4-dione (2.9 mL, 27.9 mmol, 1.3 eq.) and NaOAc (5.3 g, 64.4 mmol, 3.0 eq.) in EtOH (22 mL) and water (13 mL). The mixture was stirred for 1 h and the resulting yellow precipitate was collected via vacuum filtration. After washing with water, water/EtOH (1:1) and hexane the yielded solid was dried under vacuum affording **9** (3.17 g, 15.5 mmol, 73%).

The spectra corresponds to the published data.[3]

^{1}H NMR (400 MHz, $CDCl_3$) δ 14.67 (s, 1H), 7.44 – 7.38 (m, 4H), 7.24 – 7.15 (m, 1H), 2.59 (s, 3H), 2.48 (s, 3H) ppm.

Hydrazine monohydrate (0.31 mL, 9.8 mmol, 1 eq.) was added to a solution of **9** (2 g, 9.8 mmol, 1 eq.) dissolved in EtOH (50 mL) and the reaction mixture was heated under reflux for 3 h. Concentration under reduced pressure yielded **10** (1.96 g, 9.8 mmol, quant) which was used without further purification.

The spectra corresponds to the published data.[3]

^{1}H NMR (400 MHz, $CDCl_3$) δ 9.71 (s, 1H), 7.84 – 7.76 (m, 2H), 7.52 – 7.43 (m, 2H), 7.43 – 7.34 (m, 1H), 2.61 (s, 6H) ppm.

Compound **10** (1.0 g, 5.0 mmol, 1 eq.) was dissolved in dry acetonitrile (50 mL) and K_2CO_3 (2.1 g, 15 mmol, 3 eq.) and 2-bromoacetate ester (1.1 g, 6.5 mmol, 1.3 eq.) were added. The heterogeneous mixture was heated under reflux for 18 h. Subsequnetly, the solvent was removed under reduced pressure, the resulting residue was dissolved in EtOAc/H_2O (1:1) and the layers were separated. The aqueous layer was extracted once with EtOAc, the combined organic layers were dried over $MgSO_4$ and the solvent was removed under vacuum. The crude

product was purified by column chromatography (SiO_2, DCM/MeOH 98:2) yielding the AAP ester as a yellow solid (1.2 g, 4.2 mmol, 85%).

The spectra corresponds to the published data.[3]

^{1}H NMR (400 MHz, $CDCl_3$) δ 7.78 – 7.74 (m, 2H), 7.44 – 7.38 (m, 2H), 7.35 – 7.29 (m, 1H), 4.76 (s, 2H), 4.19 (q, *J* = 7.2 Hz, 2H), 2.50 (s, 6H), 1.23 (t, *J* = 7.1 Hz, 3H) ppm.

The AAP ester (0.50 g, 2.1 mmol, 1 eq.) was dissolved in THF/water (2:1, 24 mL) and LiOH monohydrate (175 mg, 4.2 mmol, 2 eq.) was added. The solution was stirred for 3 h at room temperature. After removing THF under reduced pressure, the aqueous phase was washed with EtOAc. The aqueous phase was then acidified with aq. HCl (to pH = 1-2) and was extracted again with EtOAc (3 × 50 mL). The combined organic layers were washed with brine (30 mL), dried over $MgSO_4$ and concentrated under reduced pressure yielding the AAP-acid **4** as a yellow solid (435 mg, 1.9 mmol, 92%).

The spectra corresponds to the published data.[3]

^{1}H NMR (400 MHz, DMSO-d_6) δ 13.12 (s, 1H), 7.74 – 7.70 (m, 2H), 7.53 – 7.48 (m, 2H), 7.45 – 7.39 (m, 1H), 4.94 (s, 2H), 2.50 (s, 3H), 2.36 (s, 3H) ppm.

NMR Spectra

DK398

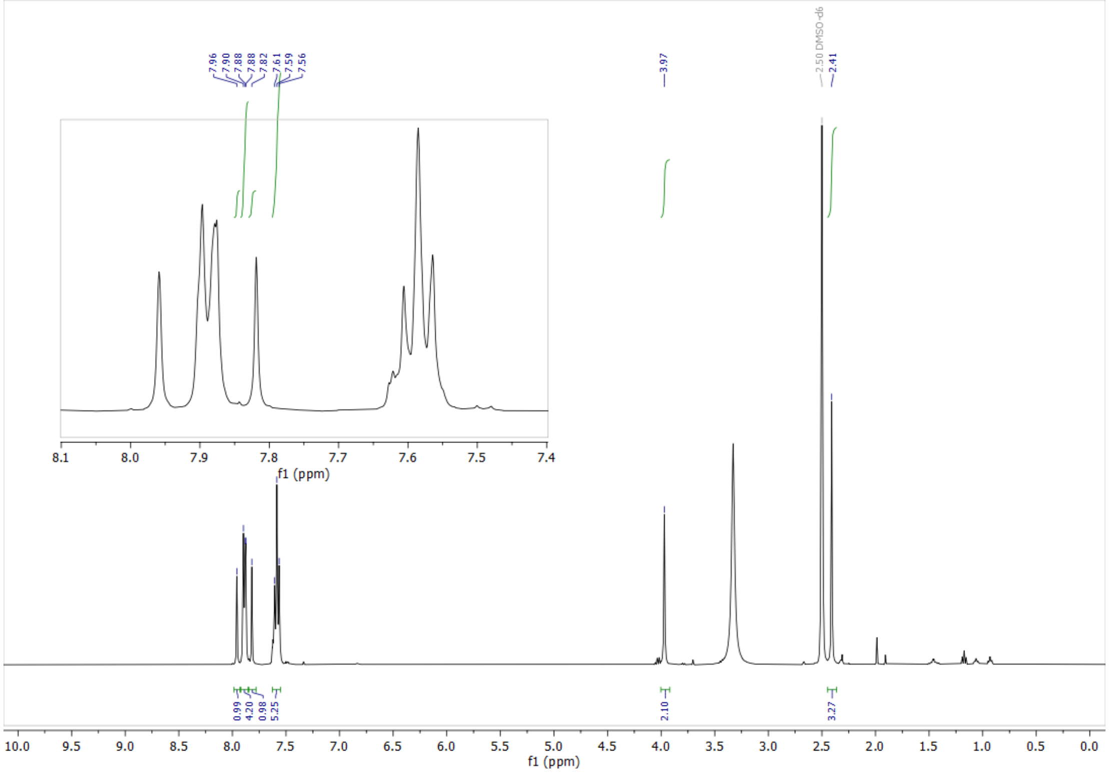

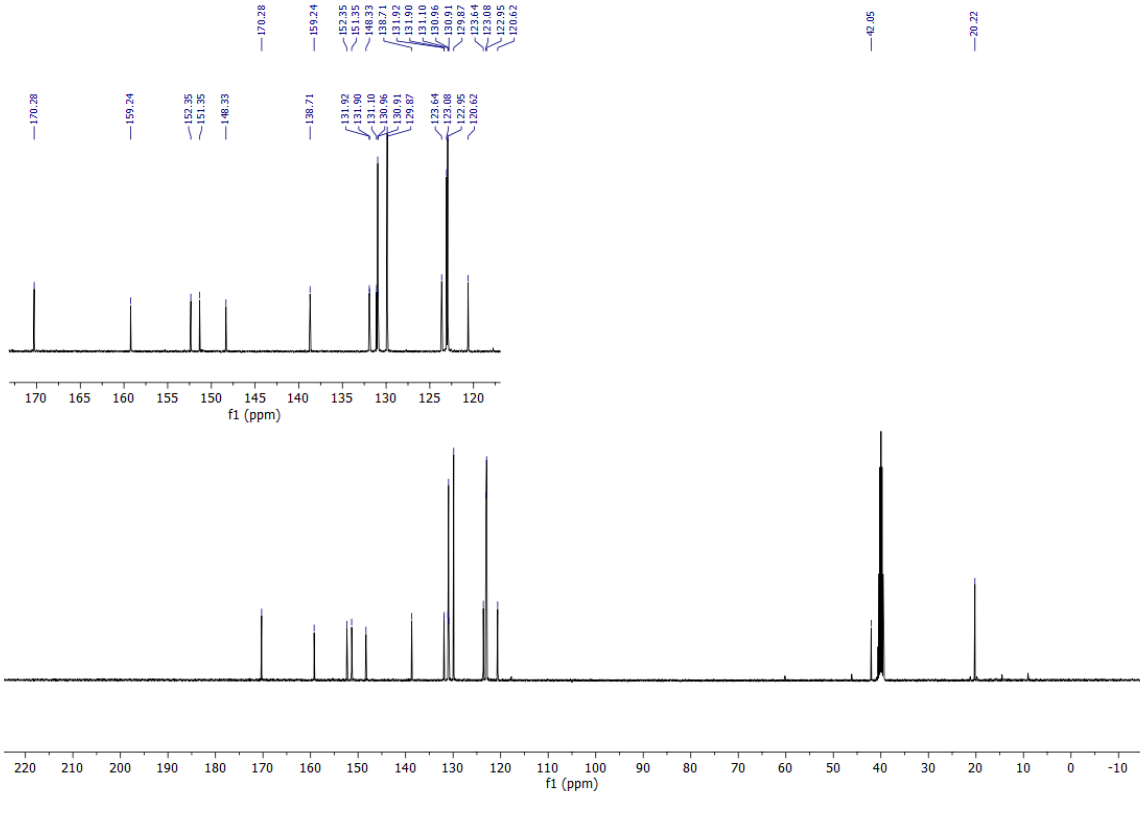

DK518

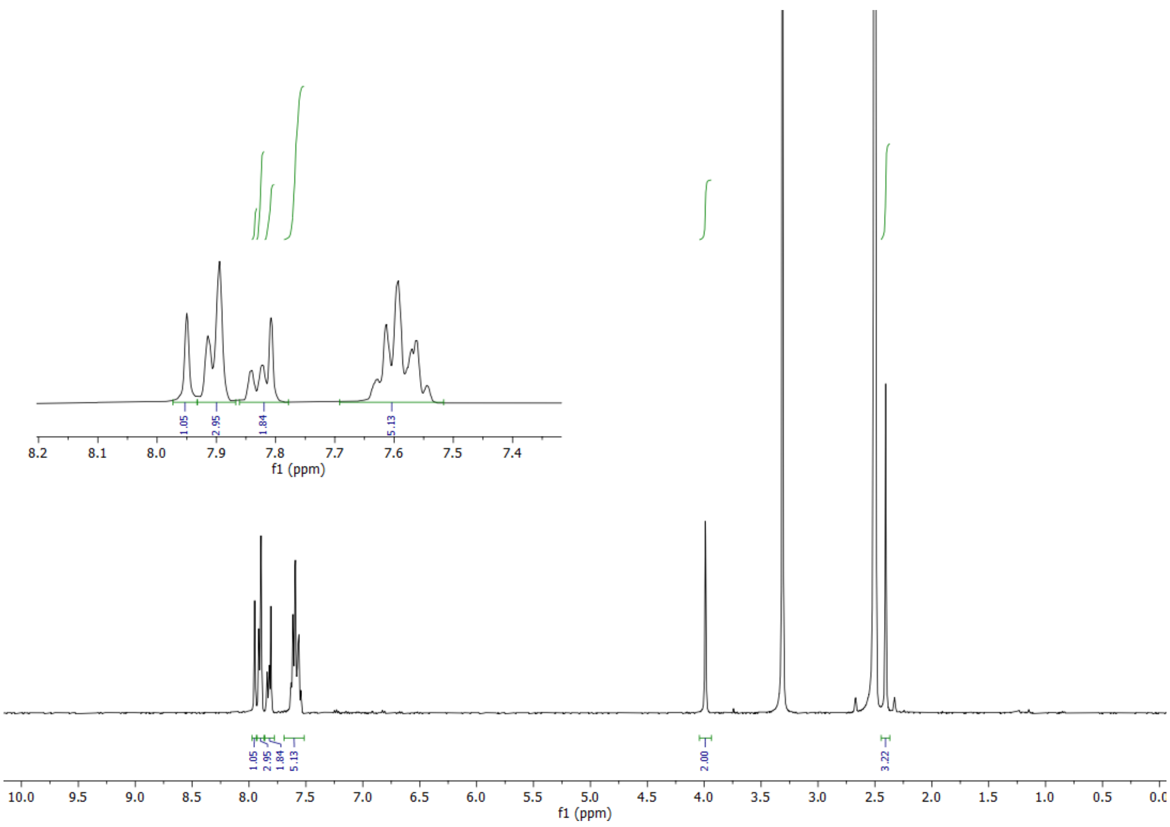
1.05
2.95
1.84
5.13
8.2 8.1 8.0 7.9 7.8 7.7 7.6 7.5 7.4
f1 (ppm)
2.00
3.22
10.0 9.5 9.0 8.5 8.0 7.5 7.0 6.5 6.0 5.5 5.0 4.5 4.0 3.5 3.0 2.5 2.0 1.5 1.0 0.5 0.0
f1 (ppm)

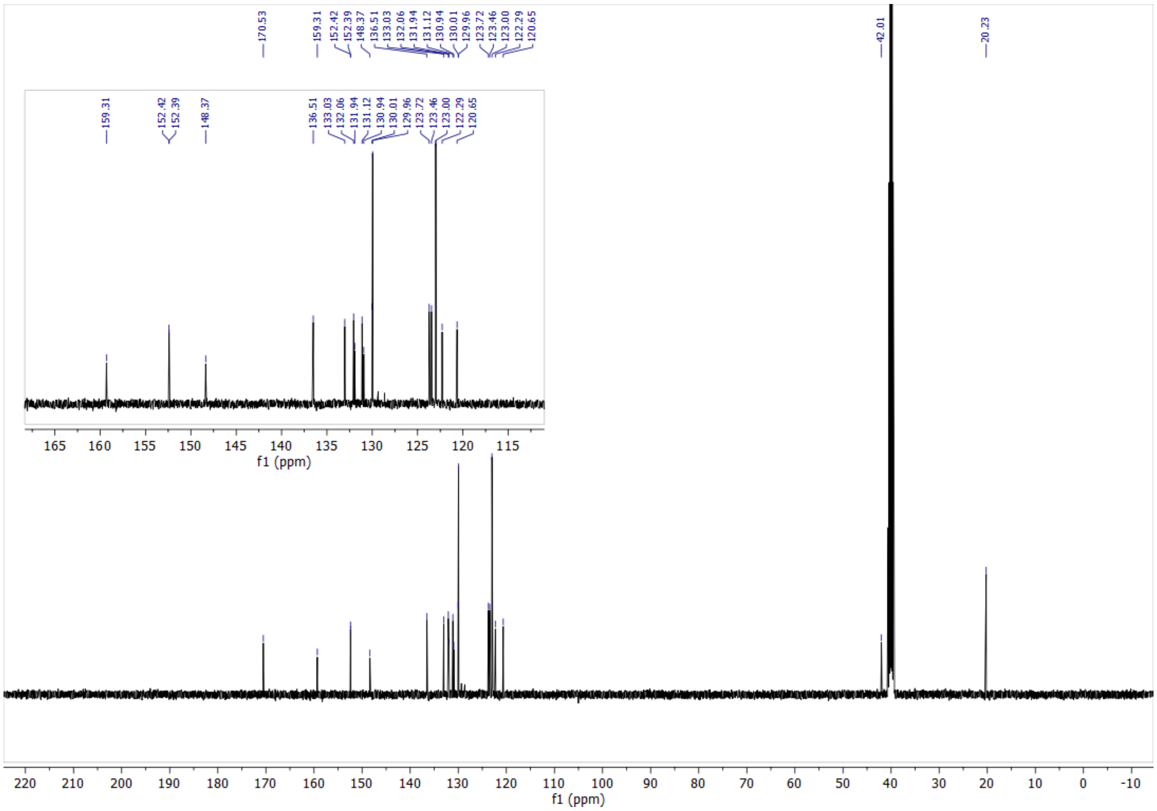
170.53
159.31
152.42
152.39
148.37
136.51
133.03
132.06
131.94
131.12
130.94
130.01
129.96
123.72
123.46
123.00
122.29
120.65
42.01
20.23
165 160 155 150 145 140 135 130 125 120 115
f1 (ppm)
220 210 200 190 180 170 160 150 140 130 120 110 100 90 80 70 60 50 40 30 20 10 0 -10
f1 (ppm)

DK557

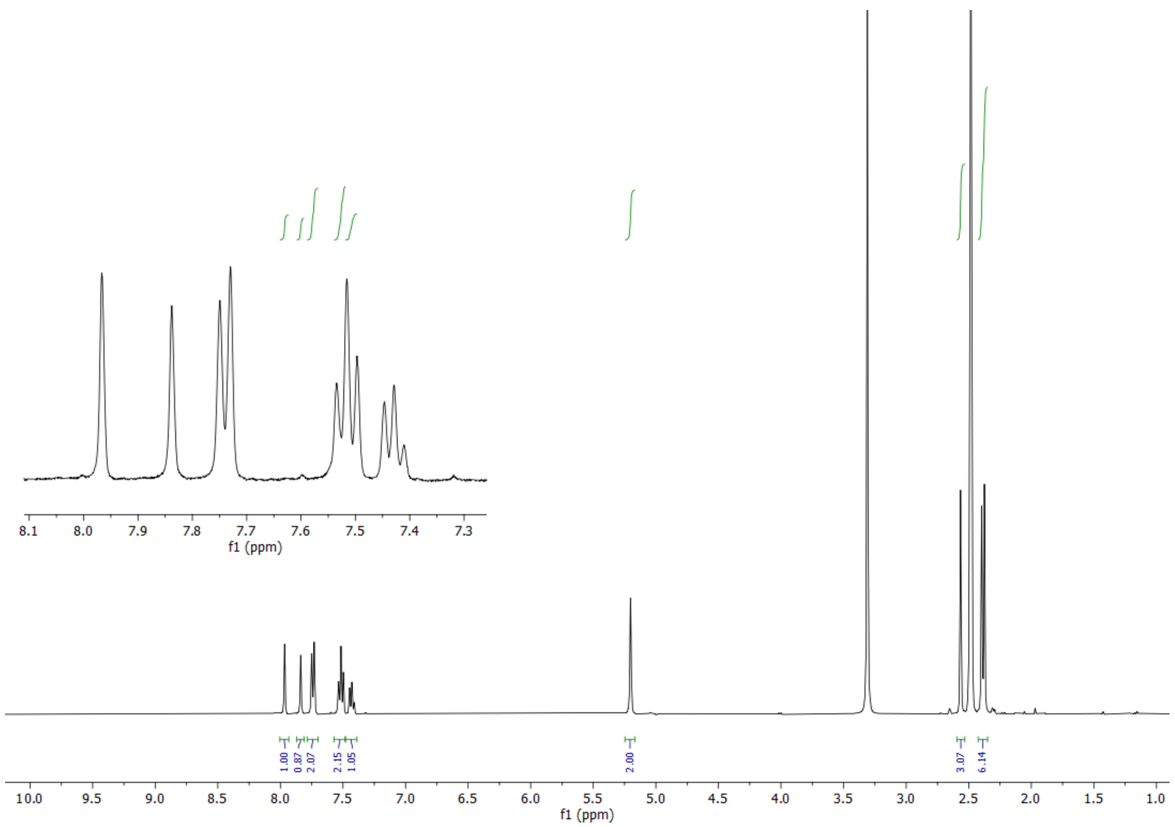

1.00
0.87
2.07
2.15
1.05
2.00
3.07
6.14
f1 (ppm)

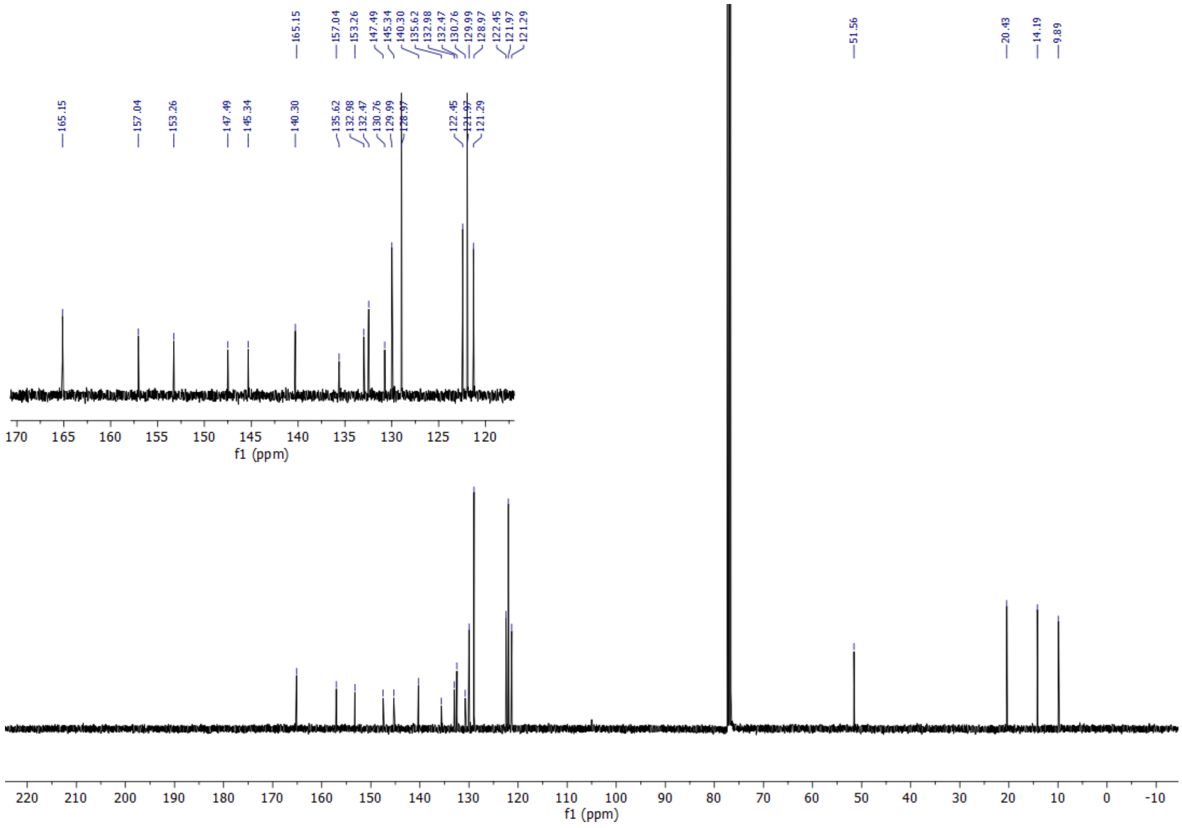

165.15
157.04
153.26
147.49
145.34
140.30
135.62
132.98
132.47
130.76
129.99
128.97
122.45
121.97
121.29
51.56
20.43
14.19
9.89
f1 (ppm)

Compound **2**

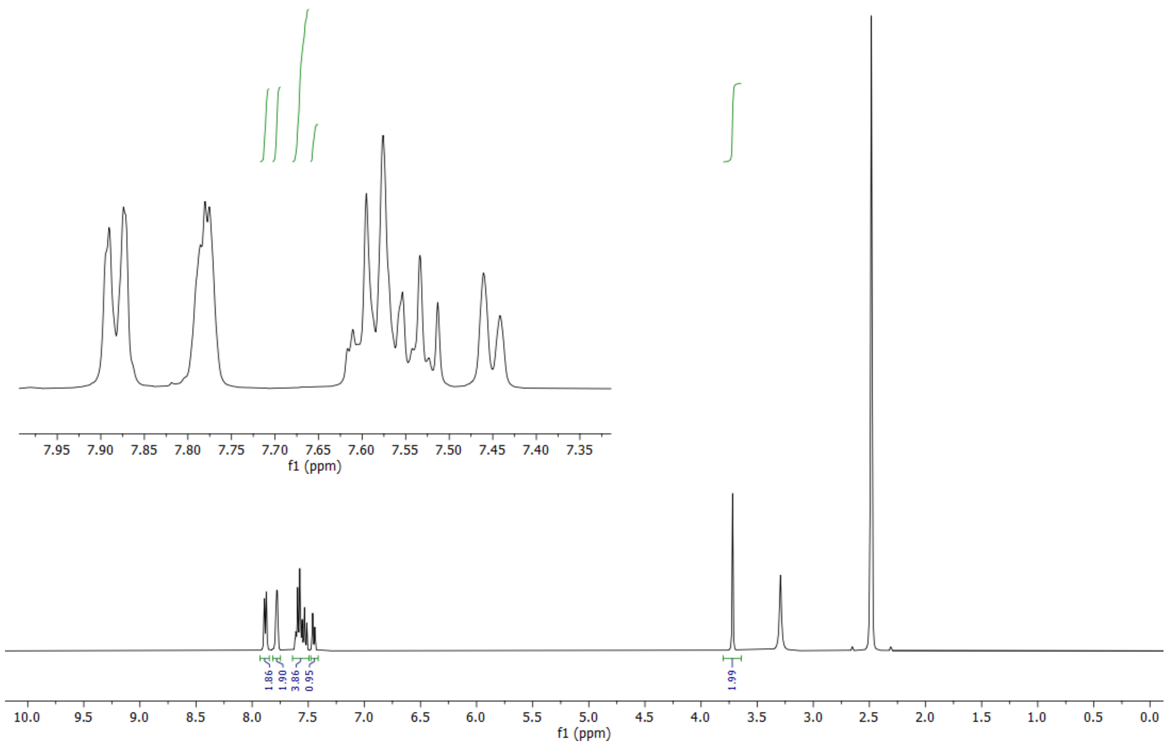
1.86
1.90
3.86
0.95
1.99
f1 (ppm)

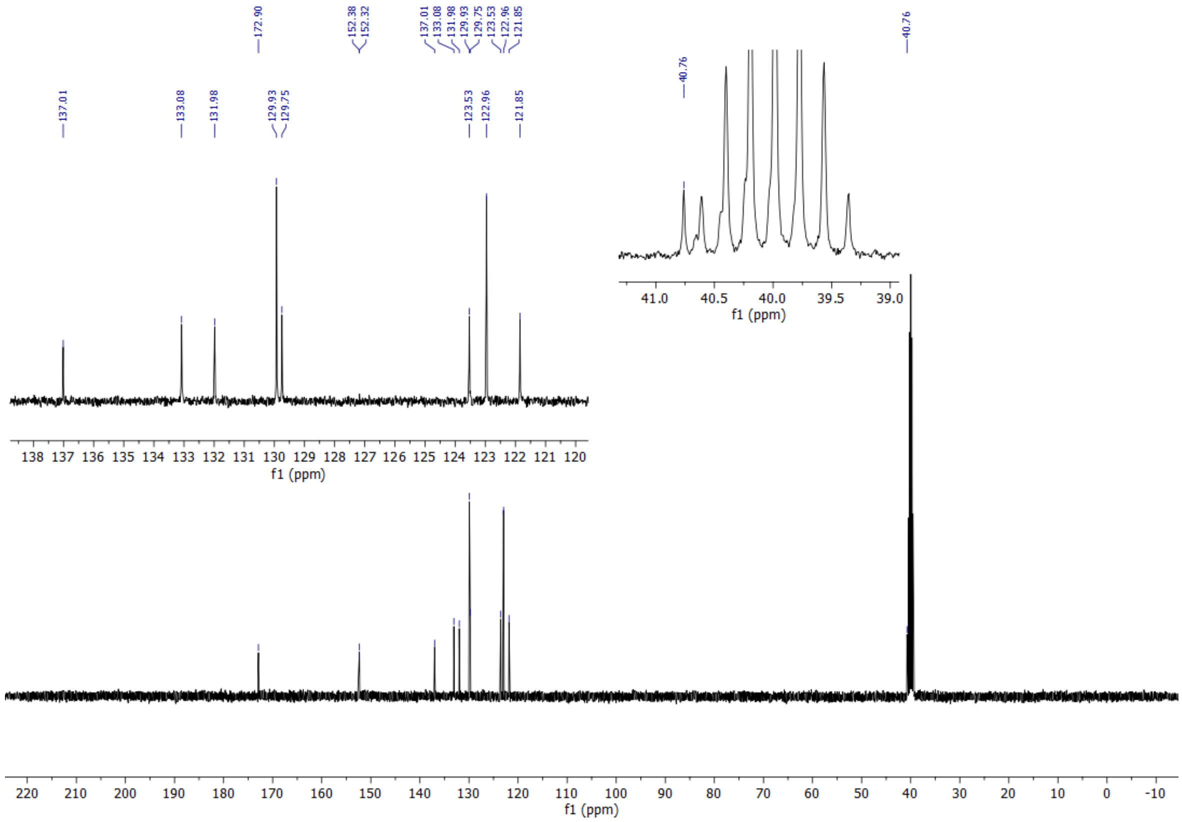
172.90
152.38
152.32
137.01
133.08
131.98
129.93
129.75
123.53
122.96
121.85
40.76
f1 (ppm)

UV-Vis absorption spectra

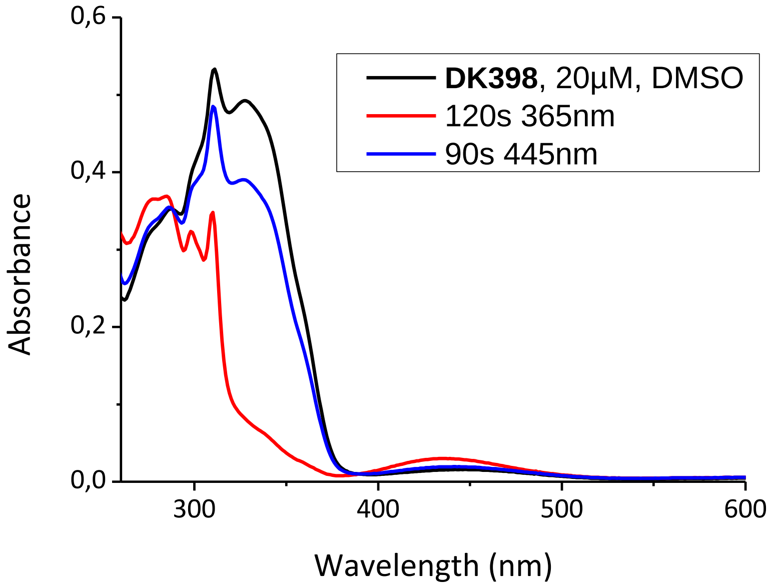

Figure S1. UV-Vis absorption spectra of **DK398** (20 µM, DMSO, 25 °C), thermally adapted and irradiated (λ = 365 and 445 nm).

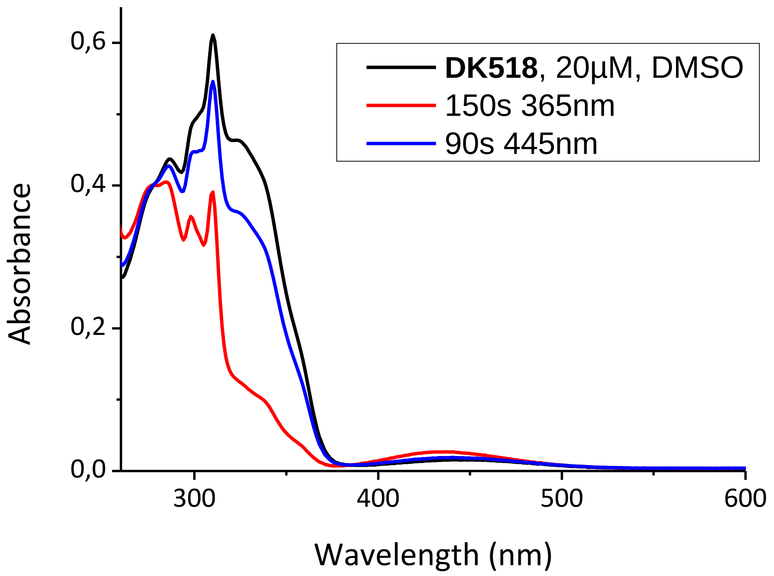

Figure S2. UV-Vis absorption spectra of **DK518** (20 µM, DMSO, 25 °C), thermally adapted and irradiated (λ = 365 and 445 nm).

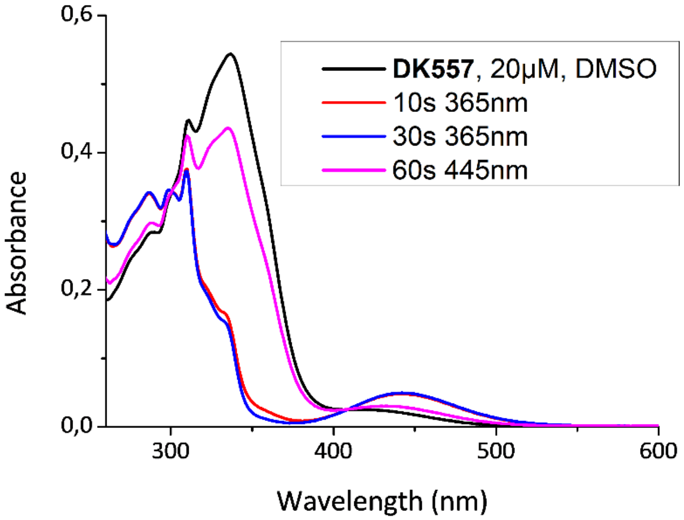

Figure S3. UV-Vis absorption spectra of **DK557** (20 µM, DMSO, 25 °C), thermally adapted and irradiated (λ = 365 and 445 nm).

Half-life measurements

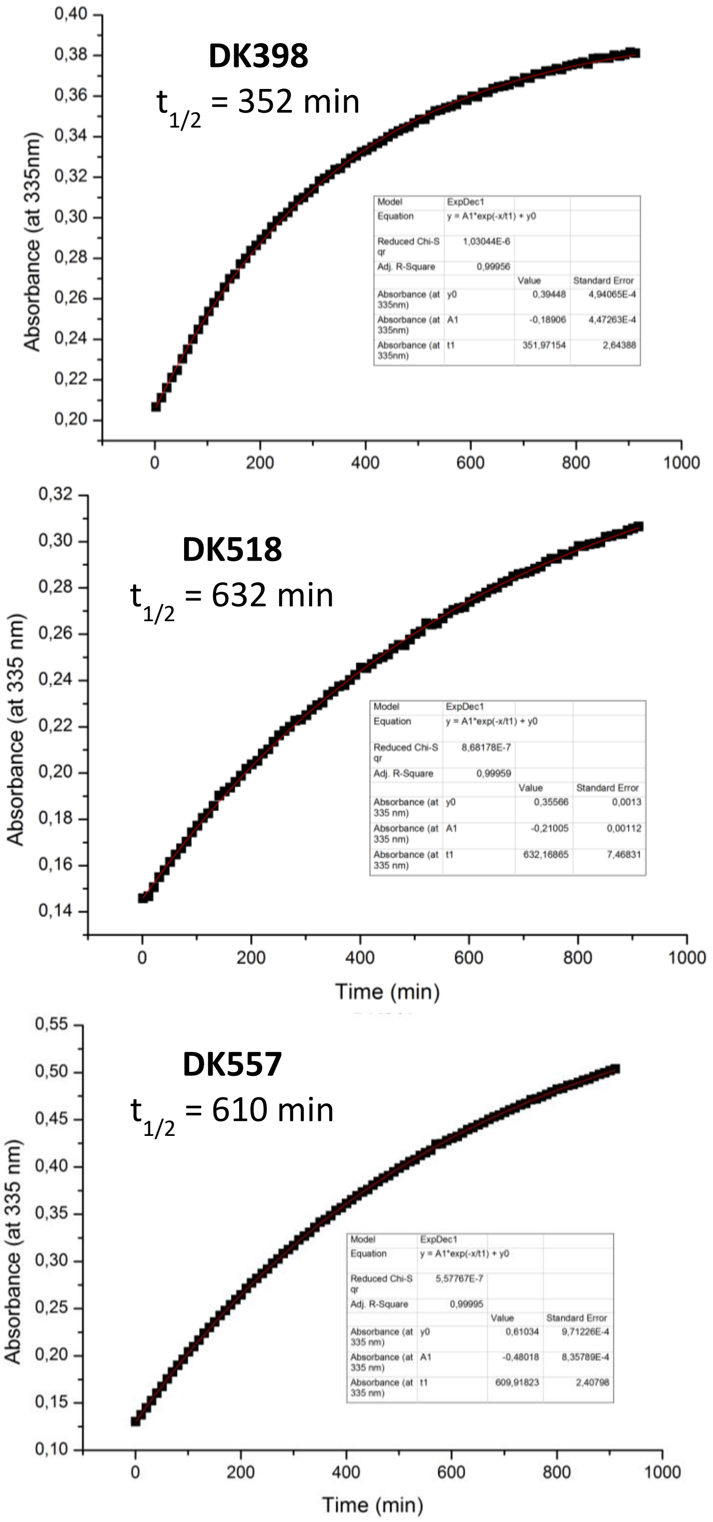

Figure S4. Rate of the dark, thermal *cis*-to-*trans* isomerization of **DK398**, **DK518** and **DK557** in kinase assay buffer (20 µM, 1% DMSO, 30 °C). Reported are half-life values for the back-isomerization reaction.

Photostationary state isomer distribution (PSD)

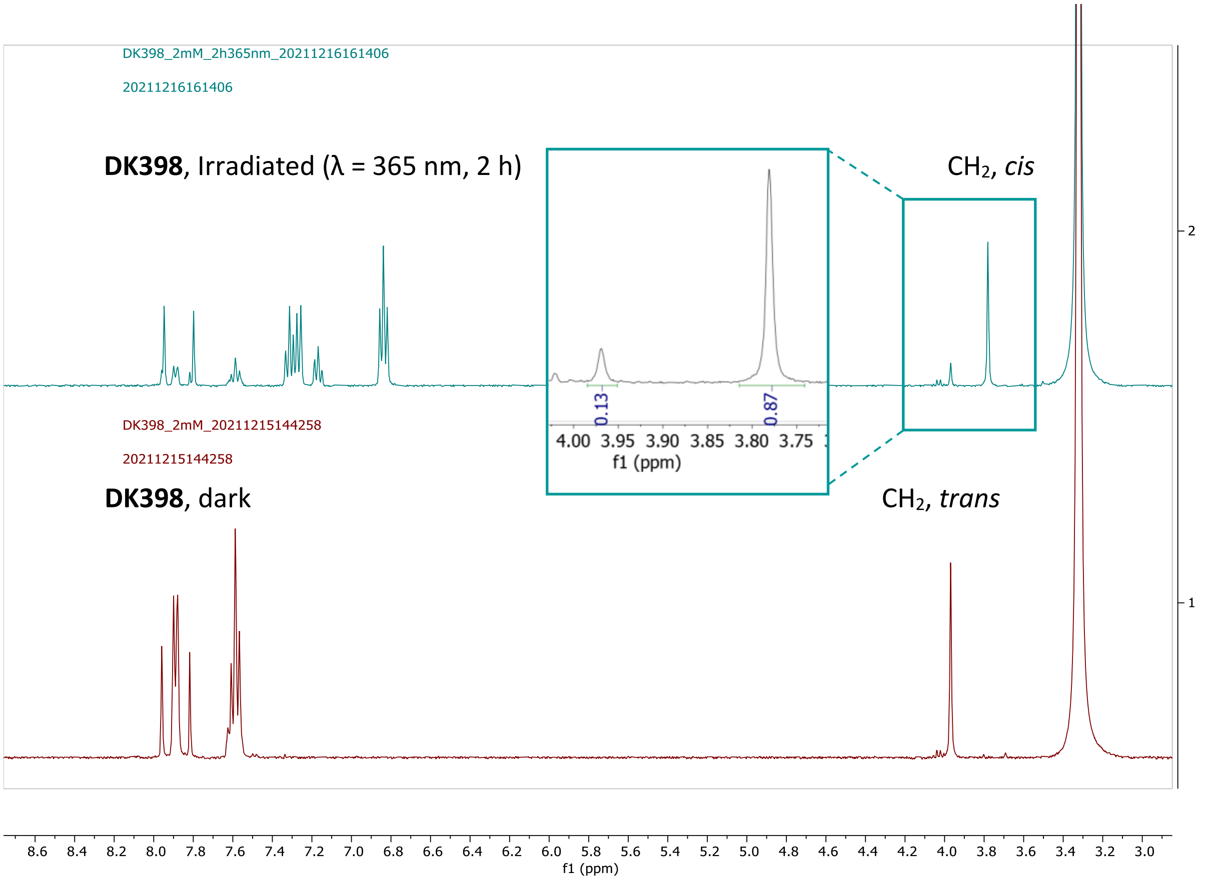

Figure S5. ^{1}H-NMR spectra of the same sample containing **DK398** (2 mM, DMSO-d_6) with measurements taken after thermal adaptation (bottom, red) and 2 h irradiation (λ = 365 nm, top, green). Isomer distribution was determined to be 87:13 *cis*/*trans* by CH_2 signal integration (in the box).

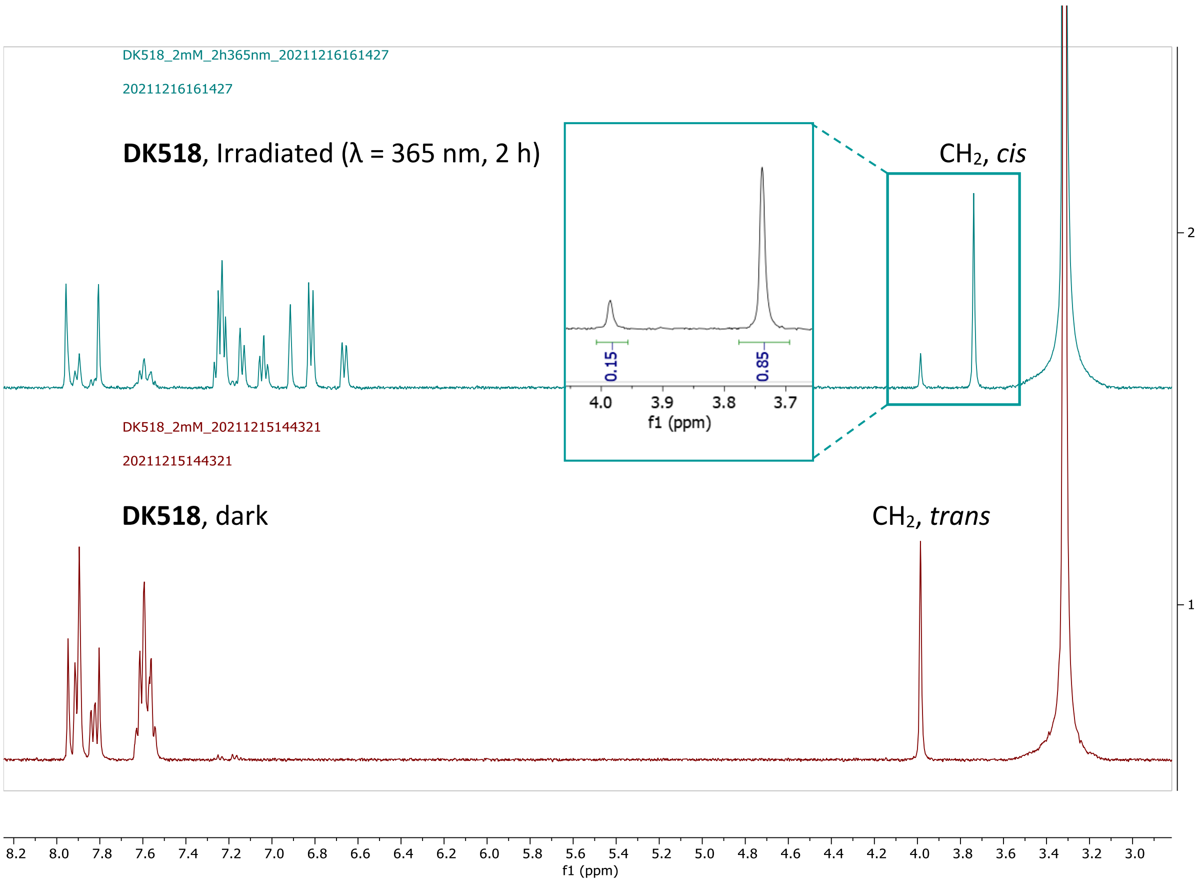

Figure S6. ^{1}H-NMR spectra of the same sample containing **DK518** (2 mM, DMSO-d_6) with measurements taken after thermal adaptation (bottom, red) and 2 h irradiation (λ = 365 nm, top, green). Isomer distribution was determined to be 85:15 *cis*/*trans* by CH_2 signal integration (in box).

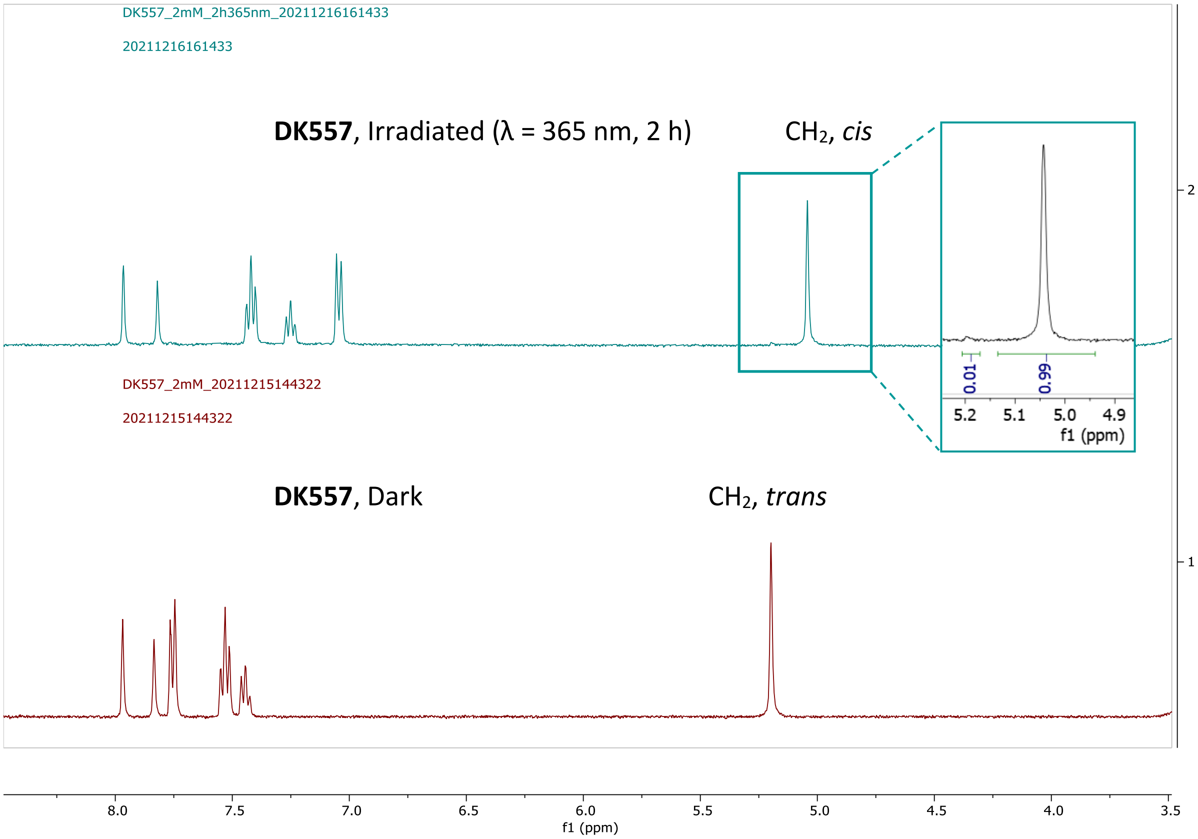

Figure S7. ^{1}H-NMR spectra of the same sample containing **DK557** (2 mM, DMSO-d$_6$) with measurements taken after thermal adaptation (bottom, red) and 2 h irradiation (λ = 365 nm, top, green). Isomer distribution was determined to be 99:1 *cis*/*trans* by CH_2 signal integration (in box).

Reductive stability (DTT)

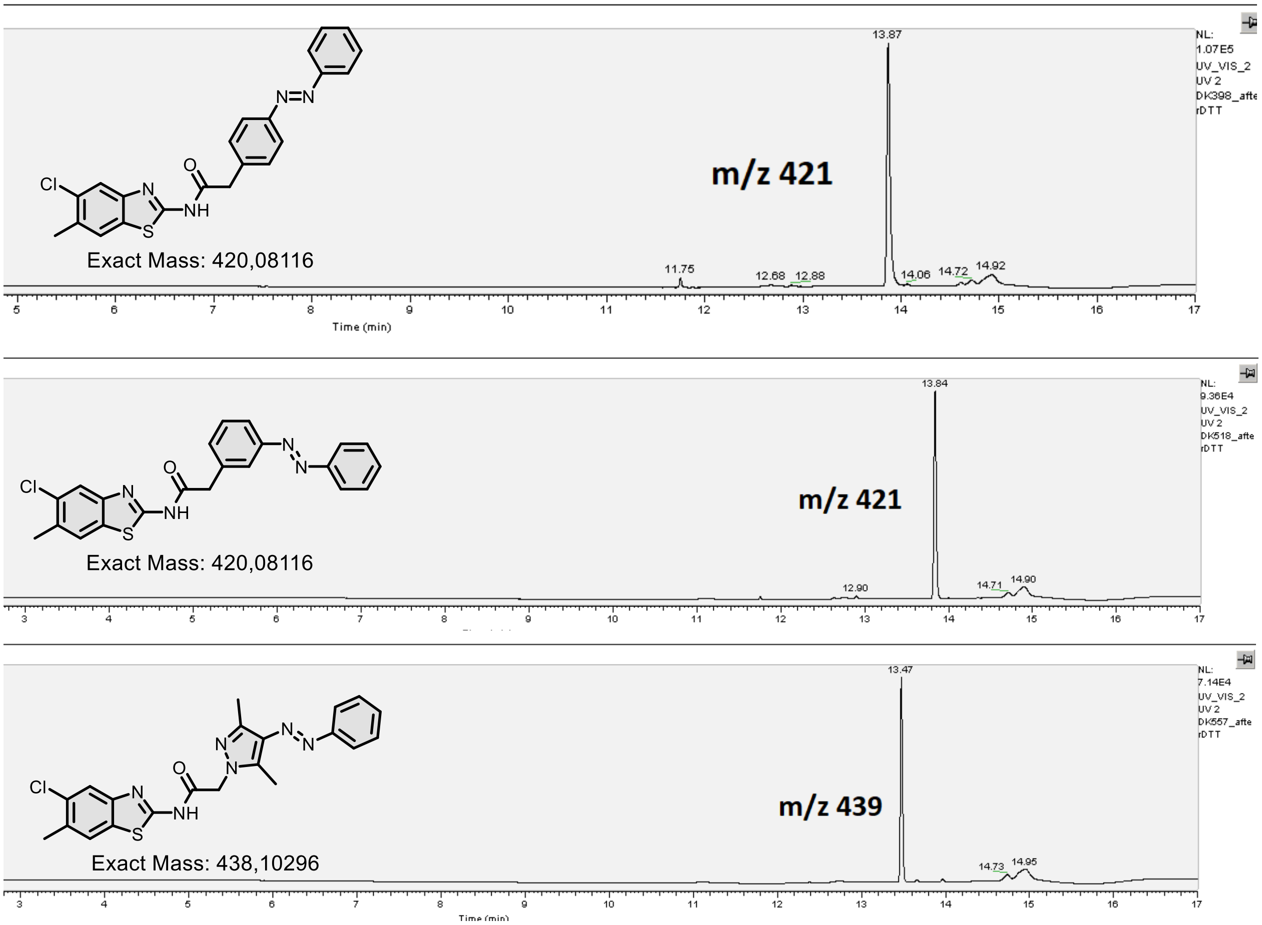

Figure S8. UV-traces (λ = 312 nm) of UPLC-MS measurements after incubation of **DK518**, **DK398** and **DK557** in buffer (10 µM, 0.5 mM DTT, 10 mM $MgCl_2$, pH 7.4) for 2h at 30 °C. Traces show the presence of **DK518**, **DK398** and **DK557**. No reduction products (hydrazine or aniline) were observed.

Irradiation effect on CK1δ activity

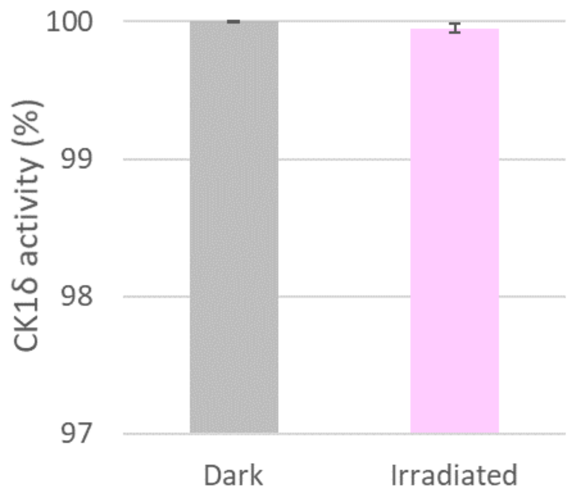

Figure S9. Effect of UV-light irradiation for the full duration of the assay (λ = 365 nm, 2h) on CK1δ activity. Shown is the relative activity of the irradiated samples compared to the 'dark' samples (100%). Reported are averages and SD values of triplicate measurements.

Molecular Docking

High-resolution crystal structures of CK1δ with no mutated residues in the entire protein and no missing residues in the active site were selected. The individual chains within a crystal structure were aligned and considered identical only if the root mean square deviation (RMSD) of the binding site residues (residue numbers: 15, 16, 23, 36, 37, 38, 56, 68, 80-91, 132, 135, 148 and 149) was less than 3 Å; otherwise, individual chains were treated as separate entities for the investigations. This resulted in 19 individual chains, which were further aligned and clustered based on the RMSD of the C-alpha atoms of the above-mentioned binding site residues using the GROMACS clustering tool (gmx cluster).[4] The clustering analysis resulted in 7 structures (4KBK:B, 4TN6:A, 4TN6:B, 6HMP:A, 6PXO:A, 6RCH:B, 6RU6:A) representing different clusters. All 7 protein structures were prepared using the Protein Prep wizard (Schrödinger Release 2020-4: Protein Preparation Wizard; Epik, Schrödinger, LLC, New York, NY, 2020). Protein preparation included the addition of missing residues and hydrogens, optimization of hydrogen bonds, removal of water molecules, and energy minimization with convergence to a maximum RMSD of 0.3 Å. The energy minimized protein structures were then processed in the Glide module (Schrödinger Release 2020-4: Glide, Schrödinger, LLC, New York, NY, 2020) to generate grids with the centroid defined by the above-mentioned binding site residues.

The three-dimensional structures of the ligands LH846 (single form only), DK398, DK518, DK557 in both the *trans* and *cis* forms were generated and optimized at pH 7.0 ± 0.5 employing LigPrep with Epik (Schrödinger Release 2020-4: LigPrep, Schrödinger, LLC, New York, NY, 2020). The processed protein structures with the grid and the ligands were subjected to molecular docking in the Glide module at extra-precision (XP) mode.[5] Kinase inhibitors binding in the ATP binding site typically form hydrogen bond interactions with the backbone amide hydrogen and oxygen of the hinge residues. Therefore, the docked pose of the ligand that displayed at least one hydrogen bond with the backbone of Leu85 and with the highest Glide score was selected for further analysis. The images of the docked poses (figures S10-S12) were obtained with the Maestro module (Schrödinger Release 2020-4: Maestro, Schrödinger, LLC, New York, NY, 2020).

Molecular Dynamics Simulations and Binding Energy Calculation

To analyze the stability of the protein-ligand complexes and estimate the binding free energies of the ligands, molecular dynamics simulations of the selected complexes were performed using the Desmond module[6] (Schrödinger Release 2020-4: Desmond Molecular Dynamics System, D. E. Shaw Research, New York, NY, 2020. Maestro-Desmond Interoperability Tools, Schrödinger, New York, NY, 2020). Each of the selected protein-ligand complexes was simulated in a truncated octahedron box solvated with explicit TIP3P water molecules. OPLS3e force field[7] was used to model the protein and photoswitchable molecules. The default energy minimization and equilibration settings of Desmond were applied to the simulation system. The equilibrated system was used for the final production run of 20 ns using the NPT ensemble at 310 K and 1 atm pressure.

The stability of the protein-ligand complexes was analyzed using the "simulation integration diagram" and "simulation quality analysis" tools of Desmond. In addition, ensemble-average Molecular Mechanics-Generalized Born Surface Area (MM-GBSA) rescoring was performed using the Prime module

(Schrödinger Release 2020-4: Prime, Schrödinger, LLC, New York, NY, 2020) to compute the relative binding free energy of the ligand molecules. Twenty equally spaced frames were extracted from the last 2.5 ns of the trajectory for each protein-ligand complex and individually used for the MM-GBSA rescoring.

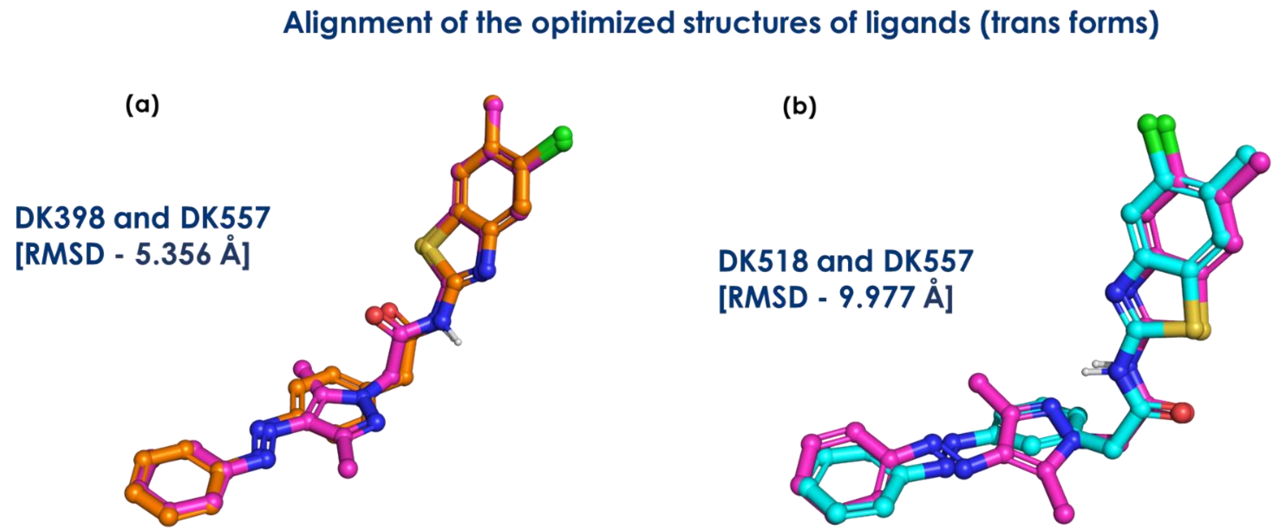

Figure S10. Superimposition of photoswitchable molecules. a) Superimposition of **DK398** (Orange) and **DK557** (Pink) (RMSD 5.356 Å) b) Superimposition of **DK518** (Cyan) and **DK557** (Pink) (RMSD 9.977 Å). **DK398**, **DK518** and **DK557** are shown as orange, cyan and pink sticks respectively

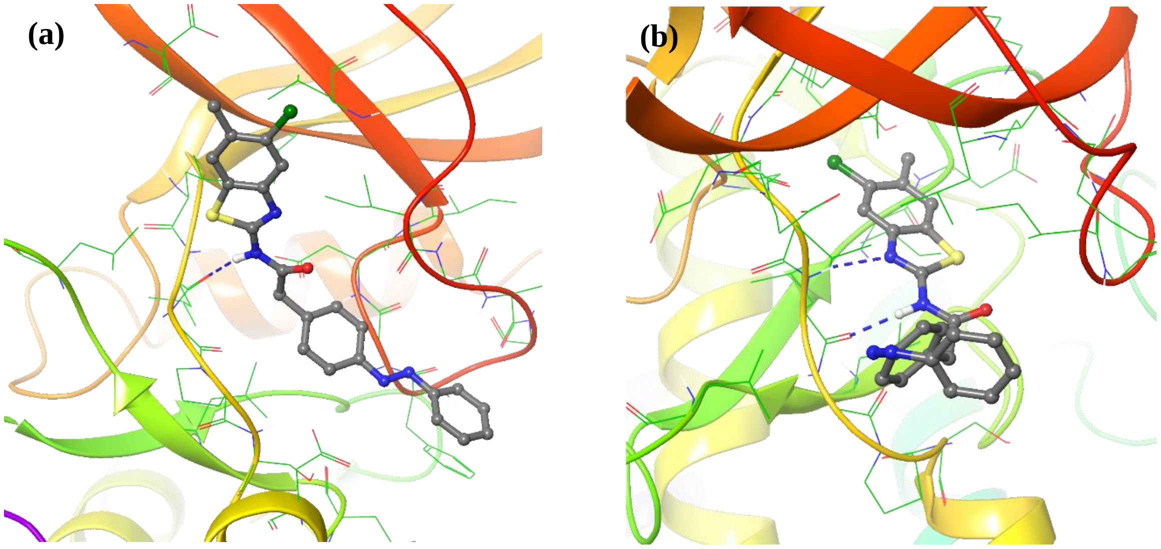

Figure S11. Best docked pose of (a) *trans* and (b) *cis* forms of **DK398** with CK1δ generated using Glide XP docking. CK1δ is shown in cartoon representation while the ligands are shown in grey ball and stick representation. The active site residues are represented in green lines and hydrogen bond interactions between the ligand and Leu85 of CK1δ are shown as blue-dotted lines.

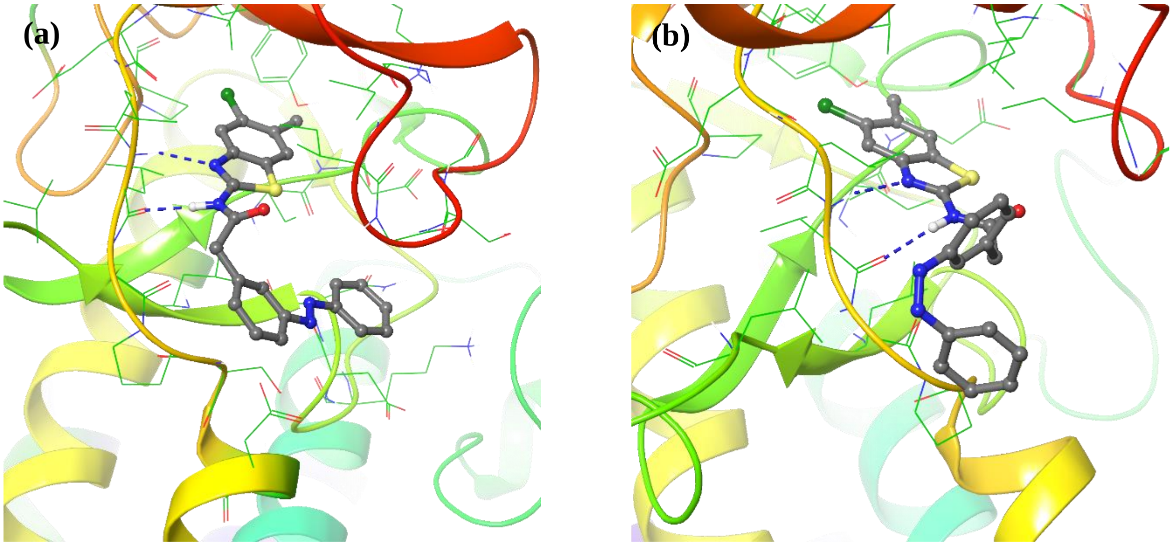

Figure S12. Best docked pose of (a) *trans* and (b) *cis* forms of **DK518** with CK1δ generated using Glide XP docking. CK1δ is shown in cartoon representation while the ligands are shown in grey ball and stick representation. The active site residues are represented in green lines and hydrogen bond interactions between the ligand and Leu85 of CK1δ are shown as blue-dotted lines.

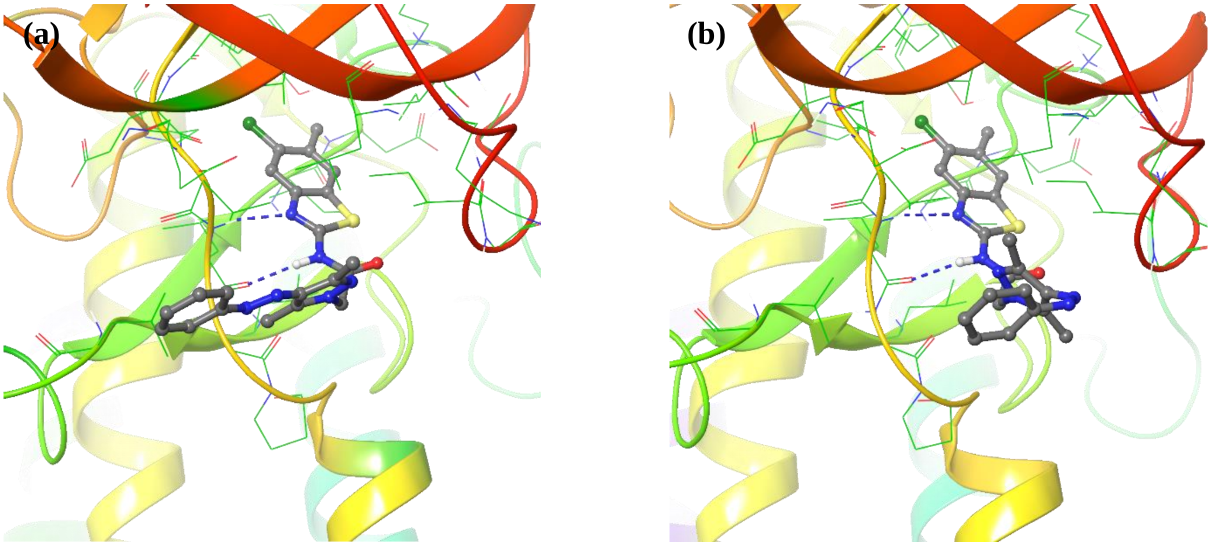

Figure S13. Best docked pose of (a) *trans* and (b) *cis* forms of **DK557** with CK1δ generated using Glide XP docking. CK1δ is shown in cartoon representation while the ligands are shown in grey ball and stick representation. The active site residues are represented in green lines and hydrogen bond interactions between the ligand and Leu85 of CK1δ are shown as blue-dotted lines.

Parametric tests

α=0.05; null hypothesis: there is no significant difference in mean between groups

	Groups considered for the test					
	DK518 *trans*	DK518 *cis*	DK557 *trans*	DK557 *cis*	p-value	Rejection of null hypothesis (yes/no)
ANOVA	☑	☑	☑	☑	4.23E-22	yes
t test 1	☑	☑	-	-	0.68 (two-tail)	no
t test 2	-	-	☑	☑	1.53E-07 (two-tail)	yes

Non-parametric tests

α=0.05; null hypothesis: there is no significant difference in median between groups

	Groups considered for the test					
	DK518 *trans*	DK518 *cis*	DK557 *trans*	DK557 *cis*	p-value	Rejection of null hypothesis (yes/no)
Kruskal-wallis	☑	☑	☑	☑	<0.001	yes
U test 1	☑	☑	-	-	0.74	no
U test 2	-	-	☑	☑	1.13E-06	yes

Figure S14. Results of parametric and non-parametric tests of the statistical difference in MM-BGSA binding energies of **DK518** and **DK557** *cis* and *trans* forms.

References

(1) Lee, J. W.; Hirota, T.; Peters, E. C.; Garcia, M.; Gonzalez, R.; Cho, C. Y.; Wu, X.; Schultz, P. G.; Kay, S. A. A Small Molecule Modulates Circadian Rhythms through Phosphorylation of the Period Protein. *Angew. Chemie Int. Ed.* **2011**, *50*, 10608–10611.

(2) Stafforst, T.; Hilvert, D.; Stafforst, T.; Hilvert, D. Modulating PNA/DNA Hybridization by Light. *Angew. Chemie Int. Ed.* **2010**, *49*, 9998–10001.

(3) Stricker, L.; Fritz, E. C.; Peterlechner, M.; Doltsinis, N. L.; Ravoo, B. J. Arylazopyrazoles as Light-Responsive Molecular Switches in Cyclodextrin-Based Supramolecular Systems. *J. Am. Chem. Soc.* **2016**, *138*, 4547-4554.

(4) Hess, B.; Kutzner, C.; Van Der Spoel, D.; Lindahl, E. GROMACS 4: Algorithms for Highly Efficient, Load-Balanced, and Scalable Molecular Simulation. *J. Chem. Theory Comput.* **2008**, *4*, 435–447.

(5) Friesner, R. A.; Murphy, R. B.; Repasky, M. P.; Frye, L. L.; Greenwood, J. R.; Halgren, T. A.; Sanschagrin, P. C.; Mainz, D. T. Extra Precision Glide: Docking and Scoring Incorporating a Model of Hydrophobic Enclosure for Protein-Ligand Complexes. *J. Med. Chem.* **2006**, *49*, 6177–6196.

(6) Bowers, K. J.; Chow, E.; Xu, H.; Dror, R. O.; Eastwood, M. P.; Gregersen, B. A.; Klepeis, J. L.; Kolossvary, I.; Moraes, M. A.; Sacerdoti, F. D.; Salmon, J. K.; Shan, Y.; Shaw, D. E. Scalable

Algorithms for Molecular Dynamics Simulations on Commodity Clusters. In *Proceedings of the 2006 ACM/IEEE conference on Supercomputing - SC '06*; ACM Press: New York, New York, USA, **2006**, 84.

(7) Roos, K.; Wu, C.; Damm, W.; Reboul, M.; M. Stevenson, J.; Lu, C.; K. Dahlgren, M.; Mondal, S.; Chen, W.; Wang, L.; Abel, R.; A. Friesner, R.; D. Harder, E. OPLS3e: Extending Force Field Coverage for Drug-Like Small Molecules. *J. Chem. Theory Comput.* **2019**, *15*, 1863–1874.
